# Inhibition of protein tyrosine phosphatase (PTP1B) and α-glucosidase by geranylated flavonoids from *Paulownia tomentosa*

**DOI:** 10.1080/14756366.2017.1368502

**Published:** 2017-09-21

**Authors:** Yeong Hun Song, Zia Uddin, Young Min Jin, Zuopeng Li, Marcus John Curtis-Long, Kwang Dong Kim, Jung Keun Cho, Ki Hun Park

**Affiliations:** a Division of Applied Life Science (BK21 plus), Institute of Agriculture and Life Science (IALS), Gyeongsang National University, Jinju, Republic of Korea;; b Department of Chemistry and Chemical Biology, Cornell University, Ithaca, NY, USA;; c Division of Applied Life Science (BK21 plus), PMBBRC, Gyeongsang National University, Jinju, Republic of Korea

**Keywords:** PTP1B, α-glucosidase, *Paulowinia tomentosa*, enzyme inhibition, kinetic analysis

## Abstract

Protein tyrosine phosphatase 1B (PTP1B) and α**-**glucosidase are important targets to treat obesity and diabetes, due to their deep correlation with insulin and leptin signalling, and glucose regulation. The methanol extract of *Paulownia tomentosa* fruits showed potent inhibition against both enzymes. Purification of this extract led to eight geranylated flavonoids (**1–8**) displaying dual inhibition of PTP1B and α**-**glucosidase. The isolated compounds were identified as flavanones (**1–5**) and dihydroflavonols (**6–8**). Inhibitory potencies of these compounds varied accordingly, but most of the compounds were highly effective against PTP1B (IC_50_ = 1.9–8.2 μM) than α**-**glucosidase (IC_50_ = 2.2–78.9 μM). Mimulone (**1**) was the most effective against PTP1B with IC_50_ = 1.9 μM, whereas 6-geranyl-3,3′,5,5′,7-pentahydroxy-4′-methoxyflavane (**8**) displayed potent inhibition against α**-**glucosidase (IC_50_ = 2.2 μM). All inhibitors showed mixed type Ι inhibition toward PTP1B, and were noncompetitive inhibitors of α-glucosidase. This mixed type behavior against PTP1B was fully demonstrated by showing a decrease in *V*_max_, an increase of *K*_m_, and *K*_ik_/*K*_iv_ ratio ranging between 2.66 and 3.69.

## Introduction

Protein tyrosine phosphatase 1B (PTP1B) is known to be a negative regulator of both insulin and leptin signalling pathways, and plays a role in glucose homeostasis and body weight regulation^1^. α-Glucosidase is a membrane bound enzyme in small intestine having a key role in carbohydrates hydrolysis. Thus PTP1B and α-glucosidase are attractive targets to treat obesity and diabetes, because these conditions are associated with increased PTP1B and α-glucosidase activities^2^. In this study we found that the methanolic extract from *paulownia tomentosa* fruits significantly inhibited both PTP1B and α-glucosidase enzymes. We proceeded to undertake a thorough kinetic analysis of the inhibition of these two enzymes by the compounds present in the extract.

Protein tyrosine phosphatase 1B (PTP1B) is a non-transmembrane phosphatase, which belongs to PTPs enzymes family and is highly expressed in the tissues targeted by insulin such as liver, muscle, and fat^3^. It catalyzes the de-phosphorylation of activated insulin receptor, and hence downregulates insulin signalling, additionally it also negatively regulates leptin signalling and contributes to obesity and metabolic disorders^4^. Moreover, high insulin sensitivity and resistance to obesity has been reported in PTP1B deficient mice undergoing through insulin and glucose tolerance tests^5^. Thus inhibition of PTP1B has been suggested as a promising approach for the treatment of type 2 diabetes (T2DM) and prevention of obesity^6^. α-Glucosidase (EC 3.2.1.20) is an exo-acting enzyme, which contributes to glycoprotein processing and carbohydrate metabolism^7^. Additionally, it speeds up the final step of carbohydrate hydrolysis, and provides high amount of intestine absorbable glucose. Therefore, α-glucosidase inhibition retards the cleavage of complex carbohydrates resulting in decreased postprandial hyperglycaemia, ultimately ameliorating complications associated with T2DM. α-Glucosidase inhibition can also greatly effect glycan structure which further affects the maturation, secretion and other important functions of glycoproteins^8^^,^^9^. Interestingly, bioactive constituents which simultaneously inhibit α-glucosidase and PTP1B enzymes display synergistic effect to antagonize hyperglycaemia and hence significantly improve insulin sensitization. So bioactive compounds with this dual inhibition profile may be promising therapeutic lead structures, which could effectively contribute in the treatment of T2DM, reduce the hyperglycaemia and suppress the accompanied hazards.

*Paulownia tomentosa* (Thunb.) Siebold & Zucc. ex Steud is a deciduous tree belonging to Paulowniaceae family, which is distributed widely in Korea, Japan and China. Phytochemical studies have revealed that a diverse array of metabolites like iridoids, lignans and flavonoids are present in this plant^10^^,^^11^. Particularly geranylated flavonoids are the major bioactive components, an observation that has attracted much attention due to their diverse biological applications.^12^ Previously multiple studies have explored the antimicrobial, cytotoxic, and antioxidant effects of these individual compounds, as well as some enzymes inhibitory activities such as targeting neuraminidase and human acetylcholinesterase have also been reported^13–15^.

In the present study the *P. tomentosa* fruits extract was characterized for their role as a source of PTP1B and α-glucosidase inhibitors. From preliminary screen we identified eight bioactive compounds, which displayed dual inhibitory functions against PTP1B and α-glucosidase. All bioactive compounds were able to inhibit both enzymes, however, their inhibitory potencies and mode of actions varied according to their skeletons. Furthermore, detailed kinetic mechanisms were fully characterized by using Lineweaver–Burk plot, Dixon plot and Yang’s method.

## Materials and methods

### Instruments and chemicals

Column chromatography was carried out with reversed phase C18 (ODS-A, 12 nm, S-150 μM, YMC), Silica gel (230–400 mesh, Merck), and Sephadex LH-20 (Pharmacia Biotech AB, Uppsala, Sweden) columns. All organic solvents used for extraction and isolation were first grade. Medium pressure liquid chromatography (MPLC) instrument was applied for separation purposes. In addition silica gel and reversed-phase cartridges purchased from Teledyne Isco were also used. TLC plates pre-coated with silica gel 60 F254 (0.25 mm, normal phase, Merck) were utilized for thin layer chromatography (TLC). Visualization of TLC plates was done by a UVGL-58 254 nm hand-held UV lamp (UVP, Cambridge, UK. ^13^C and ^1^H-NMR, and 2 D NMR experiments were acquired using a Bruker AM 500 (^1^H-NMR at 500 MHz, ^13^C-NMR at 125 MHz) spectrometer (Bruker, Karlsruhe, Germany). Different NMR solvents like CD_3_OD, CDCl_3_ and DMSO-d_6_ with TMS as internal standard (Andover, MA) were used. JEOL JMS-700 mass spectrometer (JEOL, Tokyo, Japan) was used to get EIMS and HREIMS data. Jasco J-715 CD spectropolarimeter (Gross-Umstadt, Germany) was used for measuring Circular Dichroism (CD) spectra in methanol (ca 0.1 mg/mL). Melting points were measured on an Electro Thermal 9200, UK. SpectraMax M3 multi-mode microplate reader (Molecular devices, Sunnyvale, CA) was used to measure the enzymatic hydrolysis.

### Plant material

The fruits of *P. tomentosa* were collected in July 2010, at Jinju, near Gyeongsang National University, Gyeongsangnam-do, South Korea. The sample was identified by Prof. Jae Hong Park and a voucher specimen (KHPark 071210) was deposited at the herbarium of Kyungpook National University, Daegu, South Korea.

### Extraction and isolation

The dried fruits of *P. tomentosa* (0.5 kg) were extracted with methanol (12 L) at room temperature for one week. The filtrate was concentrated to a black residue (115 g), which was washed with hexane (5 × 0.5 L) to remove oily components. After that the methanol extract (26 g) was chromatographed on silica gel (10 × 30 cm, 230–400 mesh, 720 g), eluted with a gradient of *n*-hexane and ethylacetate (20:1 to 1:2), to give ten different fractions (A–J). Fractions (D–G, 13 g) enriched with chromophoric compounds were fractionated by column chromatography on silica gel (5 × 40 cm, 800 g) and eluted with *n*-hexane to ethylacetate (50:1 to 0:1) to give 20 subtractions (A1-A20). Subfractions (A6-A15, 4.2 g) enriched with compounds (**1–8**) were fractionated through MPLC using a C18 column (130 g) with a gradient elution, using gradual increase in MeOH (0–100%) in H_2_O having flow rate of 16 ml/min, affording 40 subfractions (B1-B40). Subfractions (B12-B19, 0.56 g) containing compounds **4** and **8** were further chromatographed by MPLC using a C_18_ column, and eluted with MeOH (0–100%) in H_2_O, which yielded compounds **4** (18 mg) and **8** (26 mg). Subfractions (B21-B23, 0.38 g) enriched with compounds **6** and **7** were further chromatographed over a sephadex LH 20 column, using MeOH-H_2_O (80:20) to give compounds **6** (16 mg) and **7** (13 mg). Subfractions (B27-B35, 0.78 g) containing compounds **1**, **2**, **3**, and **5** were further chromatographed over MPLC using a C_18_ column, eluted with MeOH (0-100%) in H_2_O, which gave compounds **1** (27 mg), **2** (18 mg), **3** (23 mg) and **5** (12 mg). All isolated compounds were identified by comparison with previously reported spectroscopic data (Supplementary materials)^16^^,^^17^.

### PTP1B enzymatic assay

The PTP1B inhibitory assay was carried out using a slight modification to literature reported method^18^. *p*-nitrophenyl phosphate (*p*NPP) was used as a substrate for the measurement of enzyme activity. Buffer solution consisted of 25 mM Tris–HCl (pH 7.5), 2 mM β-mercaptoethanol, 1 mM (ethylenediaminetetraacetic acid) EDTA, and 1 mM dithiothreitol (DTT). The assay was performed by adding 10 μL test compound solution to 20 μL of enzyme (1 μg/ml), and then mixing with 40 μL of 4 mM *p*NPP in 130 μL of the given buffer using 96 well plate at 37 °C for 10 min. During the enzymatic reaction, *p*NP was produced as a result of *p*NPP dephosphorylation which was monitored by Spectra Max M3 multi-mode microplate reader at 405 nm for 30 min. NaVO_4_ was used as positive control for inhibition. The experiments were carried out in triplicate, and the samples concentration needed to inhibit 50% of enzyme activity under the assay conditions were defined as the IC_50_ values. Lineweaver–Burk and Dixon plots methods were used to determine kinetic parameters. Sigma Plot (SPCC Inc., Chicago, IL) was used to calculate these parameters.

### α-Glucosidase enzymatic assay

The α-glucosidase inhibitory activity was measured using reported experimental method with slight modifications^19^. Phosphate buffer [50 mM potassium phosphate (pH 6.8)] was used for the whole assay. *p*-nitrophenyl-α-d-glucopyranoside (pNPG) was used as substrate, whose concentration 1.2 mM was prepared in phosphate buffer. Stock solution of α-glucosidase (EC 3.2.1.20. from Baker’s Yeast) containing 0.45 units of enzyme was also prepared in phosphate buffer. DMSO was used as a solvent for dissolving the test compounds, which were then diluted to their respective concentrations. Enzymatic assay was performed by mixing 40 μl of substrate (final concentration 0.15 mM) with 10 μl of inhibitors/vehicle, 130 μl phosphate buffer and 20 μl of enzyme in 96 well plate. As a result of the catalytic reaction, *p*-nitrophenol was produced as a reaction product which was subsequently measured using spectra max M3 multi-mode microplate reader (Molecular Devices) at 37 °C, at 405 nm for 15 min. The concentrations of the compounds that inhibited 50% of enzyme activity (IC_50_) was determined by the percent inhibition ratio (percent) which was calculated accordingly: % inhibition = [(rate of control reaction – rate of sample reaction)/rate of control reaction] × 100. The experiments were performed in triplicate.

### Enzyme kinetics and progress linear determination

Lineweaver–Burk plots were used for the analysis of enzyme’s inhibitory kinetics, caused by the tested bioactive compounds, and its comparison was performed with the data collected in the absence of inhibitors. Steady-state rates were determined at different inhibitor concentrations and at changing concentrations of substrate, then enzyme inhibition mechanism and related kinetic parameters were determined. Where applicable, *K*_I_ and *K*_IS_, the two inhibition constants, when inhibitor binds with either free enzyme or the complex of enzyme–substrate, were derived from secondary plots of the slopes of the straight lines and vertical intercept (1/*V*^app^_max_), versus inhibitors concentrations. Equations (1)–(3) are the representatives equations for derivation of *K*_I_ and *K*_IS_, respectively^20^.

(1) $$\text{1}/V=K_{\text{m}}/V_{\text{max}}(\text{1}+\left[\text{I}\right]/K_{\text{I}})\text{ 1}/\text{S}+\text{1}/V_{\text{max}})$$

(2) $$\text{Slope}=K_{\text{m}}/K_{\text{I}}V_{\text{max}}\left[\text{I}\right]+K_{\text{m}}/V_{\text{max}}$$

(3) $$\text{Intercept}=\text{1}/K_{\text{IS}}V_{\text{max}}\left[\text{I}\right]+\text{1}/V_{\text{max}}$$

To determine enzyme inhibition parameters, an experiment was performed having substrate concentrations range (*p*NPP; 0.4–1.6 mM, *p*NPG; 0.075–0.3 mM), and different concentrations of tested compound, as indicated. To find out each curve parameter, a nonlinear regression program was used for data analysis using sigma Plot. Similarly *K*_m_ and *V*_max_ were derived from Lineweaver–Burk plots. *K*_ik_ and *K*_iv_ rate constants were calculated according to Equations (4) and (5) proposed by Yang’s method^21^. While Excel was used for linear regression analysis.

(4) $$K_{\text{m}}=K_{\text{m}}{,}_{0}\times \left(\text{1}+\left[\text{I}\right]/K_{\text{ik}}\right)$$

(5) $$V_{\text{m}}=V_{\text{m}}{,}_{0}\times \left(\text{1}+\left[\text{I}\right]/K_{\text{iv}}\right)$$

### Statistical analysis

All measurements were made in at least triplicate. The results were subject to variance analysis using sigma plot. Differences were considered significant at *p* < .05.

## Results and discussion

### Separation and characterization of PTP1B and α-glucosidase inhibitors

**Figure 1.**
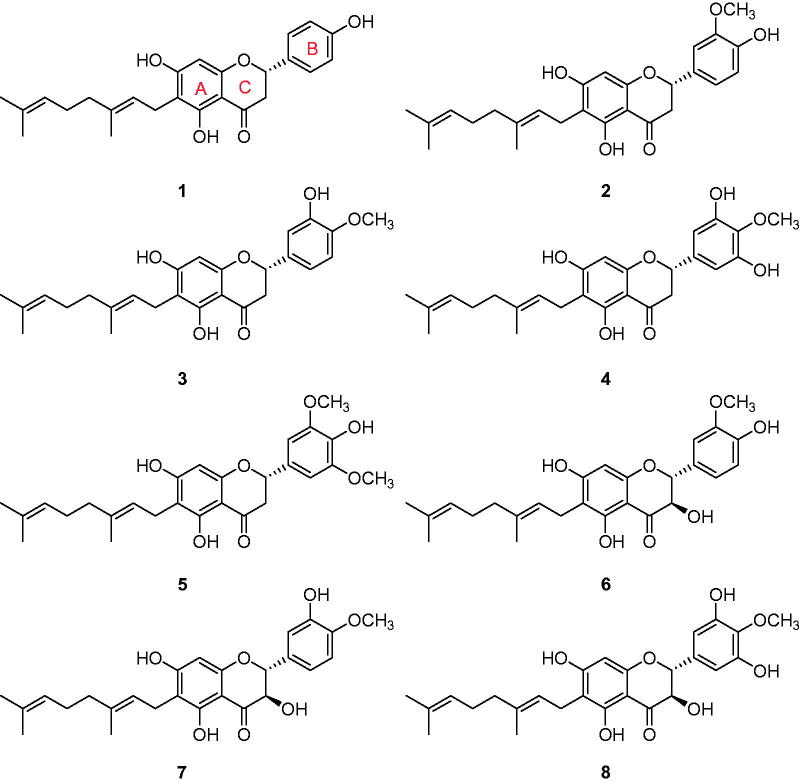
Chemical structures of isolated geranyl compounds (**1**–**8)** from the fruits of *P. tomentosa*.

The extracts from different solvents (water, methanol, chloroform) were examined for their enzymatic inhibitory activities against PTP1B and α-glucosidase. The enzymes were screened according to standard literature procedures by following the hydrolysis of *p*-nitrophenyl phosphate for PTP1B and *p*-nitrophenyl glucopyranoside for α-glucosidase spectrophotometrically^18^^,^^19^. Among them, the methanol extract displayed the most potent inhibitory activity against both PTP1B (IC_50_ = 35 μg/ml) and α-glucosidase (IC_50_ = 82 μg/ml). Next we conducted phytochemical investigations to isolate compounds responsible for this inhibition. This was achieved *via* repeated column chromatography (CC) over silica gel, octadecyl-functionalized silica gel and Sephadex LH-20, these efforts led to the isolation of eight bioactive compounds. Structure identification of these compounds were carried out by spectroscopic analysis, and their comparison with published data (Supplementary materials). All compounds shared a common feature, they were adorned with a geranyl group at the C-6 position of the flavonoid skeleton. The isolated compounds were identified as mimulone (**1**), 3′-*O*-methyldiplacone (**2**), 4′-*O*-methyldiplacone (**3**), 6-geranyl-3′,5,5′,7-tetrahydroxy-4′-methoxyflavanone (**4**), 3′-*O*-Methyl-5′-*O*-methyldiplacone (**5**), 3′-*O*-methyldiplacol (**6**), 4′-*O*-methyldiplacol (**7**), and 6-geranyl-3,3′,5,5′,7-pentahydroxy-4′-methoxyflavane^16^^,^^17^ (**8**) (Figure 1).

### PTP1B inhibitory activities

**Figure 2.**
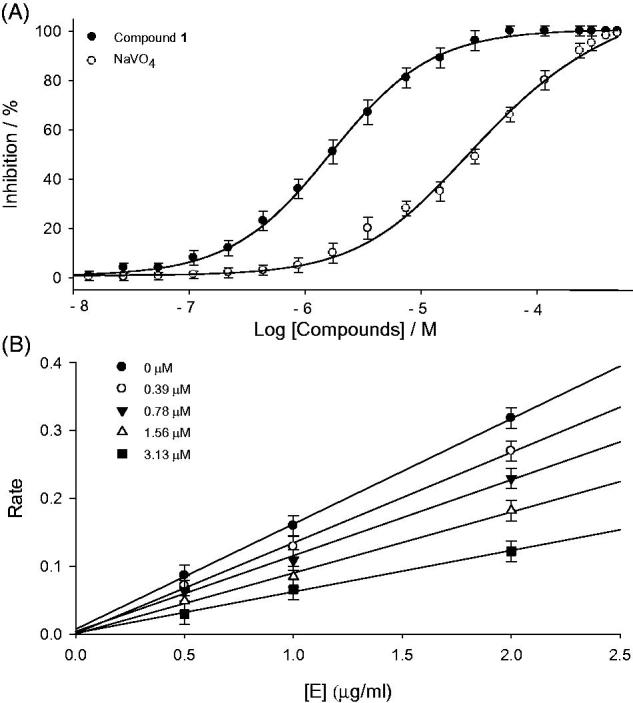
(A) Inhibitory effect of compound (**1**) and positive control (NaVO_4_) on PTP1B activity. (B) Determination of the reversible inhibitory mechanism of compound **1** on PTP1B. Data represent the results of three independent experiments performed in triplicates for each sample.

First of all, the individual isolated constituents (**1–8**) from *P. tomentosa* fruits were tested in an *in vitro* PTP1B inhibition assay, using recombinant human protein tyrosine phosphate 1B (EC 3. 1. 3. 48)^1^. All the isolated flavonoids (**1–8**) exhibited significant inhibition, having IC_50_ values in the range of 1.9–8.2 μM. However, the observed activities were slightly affected by subtle changes in compounds structures, the most effective compound was flavanone **1** (IC_50_ = 1.9 μM), whereas geranyl group was found to be a critical functionality for PTP1B inhibition. Because its mother compound, naringenin showed much lower inhibition with 130.1 μM of IC_50_^23^. Comparing with other inhibitors, the potent activity of **1** was attributable to the presence of hydroxyl group at 4′-position on B-ring: **2** (IC_50_ = 3.9 μM) vs. **4** (IC_50_ = 5.9 μM), and **5** (IC_50_ = 3.8 μM) vs. **4** (IC_50_ = 5.9 μM). Figure 2(A) shows dose-dependent inhibitory effects of the most effective compound **1** and NaVO_4_ which is a known PTP1B inhibitor and used as a positive control.

### Kinetic analysis of PTP1B inhibitors

**Figure 3.**
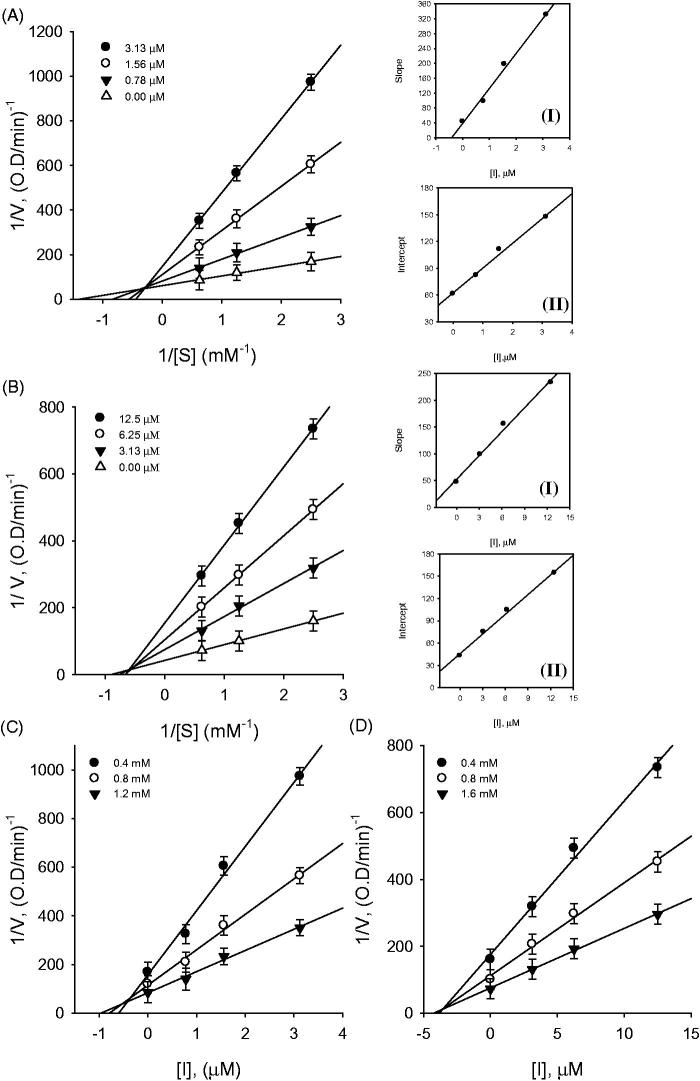
(A–D) Kinetic assays of PTP1B inhibition, caused by compounds **1** and **6**. (A and B) Lineweaver–Burk plots were constructed for the inhibition of PTP1B. The plots are expressed as 1/velocity (1/V) versus 1/substrate (1/[S]) with or without inhibitors. Insets (I) and (II) represents the secondary plots of the slopes and the intercepts of the straight lines versus concentrations of compound **1** and **6**. (C and D) Dixon plots for inhibition of PTP1B by compound **1** and **6**, respectively.

Kinetic assays were repeated in the presence of different concentrations of compounds (**1–8**) to further characterize inhibition of *p*-nitrophenylphosphate hydrolysis. The inhibition of PTP1B by the most effective compound **1** is illustrated in Figure 2(B), respectively. A plot was drawn by the initial velocity versus enzyme concentration in the presence of different concentrations of compound **1**. The slope decreased upon increasing inhibitor concentrations, indicating that compound **1** is a reversible inhibitor (Figure 2(B)). All inhibitors manifested a similar relationship between enzyme activity and concentration. The *K*_i_ values of representative compounds **1** and **6** were determined to be 0.8 μM and 3.8 μM, respectively (Figure 3(C,D)). Similarly the *K*_i_ values of all tested compounds ranged from 0.8 to 8.1 μM (Table 1).

**Table 1.** Inhibitory activities on PTP1B and α-glucosidase of isolated compounds.

	PTP1B			α-Glucosidase		
Compounds	IC_50_ (μM)^a^	*K_*i*_* (μM)^b^	Type of inhibition	IC_50_ (μM)^a^	*K_*i*_* (μM)^b^	Type of inhibition
**1**	1.9 ± 0.1	0.8 ± 0.1	Mixed Type I	30.7 ± 1.5	26.7 ± 1.4	Noncompetitive
**2**	3.9 ± 0.3	3.2 ± 0.2	Mixed Type I	18.4 ± 0.9	17.2 ± 1.0	Noncompetitive
**3**	7.8 ± 0.6	8.1 ± 0.6	Mixed Type I	19.6 ± 1.1	18.1 ± 0.9	Noncompetitive
**4**	5.9 ± 0.4	6.1 ± 0.5	Mixed Type I	6.5 ± 0.5	7.1 ± 0.6	Noncompetitive
**5**	3.8 ± 0.3	3.5 ± 0.3	Mixed Type I	78.9 ± 2.1	72.6 ± 2.3	Noncompetitive
**6**	4.9 ± 0.5	3.8 ± 0.3	Mixed Type I	17.8 ± 1.1	16.9 ± 1.2	Noncompetitive
**7**	8.2 ± 0.6	7.9 ± 0.4	Mixed Type I	25.8 ± 1.2	24.4 ± 1.3	Noncompetitive
**8**	6.6 ± 0.5	6.2 ± 0.4	Mixed Type I	2.2 ± 0.2	3.6 ± 0.3	Noncompetitive
**NaVO_**4**_**^d^	32.6 ± 1.5	NT^c^	NT^c^	>200	NT^c^	NT^c^
**Voglibose**^d^	>200	NT^c^	NT^c^	24.5 ± 1.2	NT^c^	NT^c^

a All compounds were examined in as set of experiments repeated three times; IC_50_ values of compounds represent the concentration that caused 50% enzyme activity loss.

b Values of inhibition constant.

c NT: not tested.

d Positive control.

Further kinetic analysis showed that compounds **1** and **6** exhibited a mixed type inhibition, because increasing concentrations of substrate resulted in a family of lines which shared a common intercept on the left of the vertical axis and above the horizontal axis (Figure 3(A,B)). More detailed parameters can be derived by changing the concentrations of substrate and inhibitors, followed by measurement of residual enzyme–substrate complex. This can help to specifically identify the predominant mode of inhibition, in the two possible mechanisms (Mixed type Ι and ΙΙ)^24^. In this regard parameters *K*_I_ and *K*_IS_ which reflect the affinity of compound **1** either for free enzyme or substrate bound enzyme, can be fitted to Equations (2) and (3). As a result the values of these constants, when the inhibitor binds with free enzyme (*K*_I_) and with enzyme-substrate complex (*K*_IS_) were obtained from the secondary plots of the *K*_m_ and *V*_max_ versus compound **1** concentration respectively. This analysis showed that compound **1** had the following attributes: *K*_I_ = 0.4 and *K*_IS_ = 2.2 (Figure 3 inset), which indicates that the affinity of inhibitor for free enzyme was stronger than that for enzyme-substrate complex. Thus Compound **1** was accordingly assigned as a mixed type Ι inhibitor of PTP1B. Interestingly, Figure 3(B) inset shows that compound **6** was also a mixed type Ι inhibitor.

### α-Glucosidase inhibitory activities

**Figure 4.**
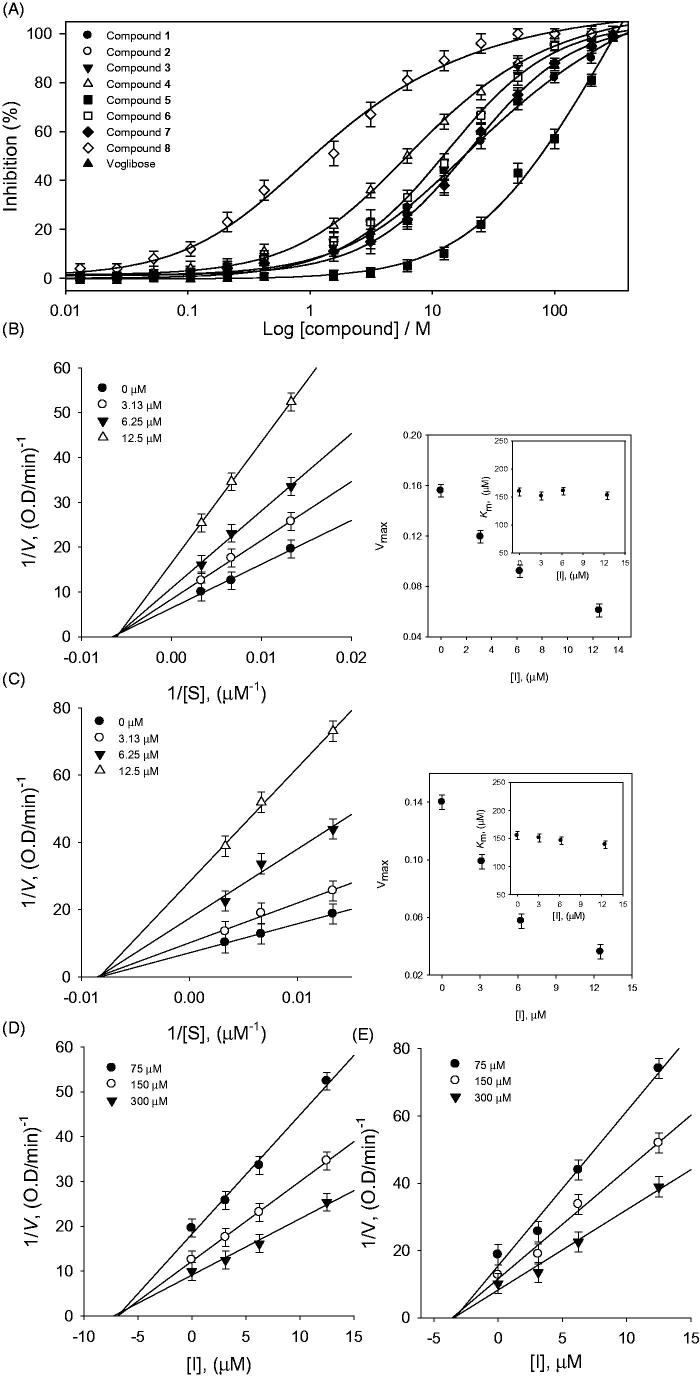
(A) Inhibitory effects of compounds (**1**–**8**) on α-glucosidase activity. (B and C) Linweaver–Burk plots of compounds **4** and **8** for the inhibition of α-glucosidase catalyzed hydrolysis of *p*-nitrophenyl glucopyranoside. (D and E) Dixon plots of inhibition of α-glucosidase by compounds **4** and **8**, respectively, which were used for determination of *K_i_* values.

Similarly the isolated flavonoids (**1–8**) were also examined for inhibitory activities against α-glucosidase. All compounds showed significant α-glucosidase inhibition with IC_50_s in the range of 2.2–78.9 μM (Figure 4(A)). Among the tested compounds the potency of compound **8** (IC_50_ = 2.2 μM) can be favourably compared with numerous sugar derived α-glucosidase inhibitors which are currently used as therapeutics, such as voglibose (IC_50_ = 24.5 μM)^22^. Our results shows some interesting facets of the SAR: it appeared that better inhibition was observed when there were two free hydroxyl groups on C-3´ and C-5´ positions in the B-ring. This can be seen by comparing compound **4** (IC_50_ = 6.5 μM) with methylated analogue **5** (IC_50_ = 78.9 μM). There was not much difference in the potencies of dihydroflavonol (**6** and **7**) and their flavanone analogues (**2** and **3**) as shown in Table 2. On the other hand dihydroflavonol **8** (IC_50_ = 2.2 μM) was more effective than its analog **4** (IC_50_ = 6.5 μM).

**Table 2.** Effect of different concentrations of compound **1** on *V*_max_, *K*_m_, and the *K*_ik_ to *K*_iv_ ratio using PTP1B and α-glucosidase.

Enzyme	[I] µM	*V*_max_	*K*_m_	*K*_ik_/*K*_iv_
PTP1B^a^	0	0.016	0.712	//
	0.78	0.012	1.193	2.660
	1.56	0.009	1.779	3.335
	3.13	0.007	2.248	3.694
α-Glucosidase^b^	0	0.157	157.68	//
	12.5	0.117	154.47	12.42
	25	0.082	153.76	19.34
	50	0.054	153.91	27.48

*V*_max_ and *K*_m_ values were calculated according to Lineweaver–Burk from the data shown in Figures 3, 4 and supplemental. *K*_ik_/*K*_iv_ ratio was calculated according to Yang et al.^21^

a *p*-Nitrophenyl phosphate (*p*NPP) as substrate.

b *p-*Nitrophenyl glucopyranoside (*p*NPG) as substrate.

### Kinetic analysis of α-glucosidase inhibitors

The α-glucosidase inhibitory properties of isolated compounds (**1–8**) were also modelled using double reciprocal plots such as Lineweaver–Burk and Dixon plots. This analysis showed that *V*_max_ decreased without changing *K*_m_ in presence of increasing concentrations of inhibitors **4** and **8** (Figure 4(B,C)). The x-intercept (−1/*K*_m_) was unaffected by inhibitor concentrations, whereas 1/*V*_max_ increased. These features indicate that compounds **4** and **8** exhibited noncompetitive inhibitory characteristics towards α-glucosidase^25^. The *K*_i_ values of these flavonoids were easily measured from Dixon plots (Table 1). The Dixon plots of compounds **4** and **8** displayed in (Figure 4(D,E)). All inhibitors showed a similar relationship between enzyme activities and enzyme concentrations (Supplementary materials).

To further confirm the inhibitory behaviour of compound **1** towards PTP1B and α-glucosidase, the results were analysed using Yang’s method^21^. According to this procedure: *K*_m_ and *V*_max_ are plotted against the inhibitor concentrations, the new kinetic constants *K*_ik_ can be fit to Equation (4)), while *K*_iv_ can be fit Equation (5). From these results of the fit, *K*_ik_/*K*_iv_ ratio of PTP1B inhibition was observed to be ranging from 2.66 to 3.69, which are further consistent with mixed type inhibition. *K*_ik_/*K*_iv_ ratio (12.42–27.48) from the kinetics of α-glucosidase accorded with noncompetitive behaviours (Table 2).

## Conclusions

This study demonstrated that the methanol extract of *P. tomentosa* fruits showed potent inhibitory activity towards PTP1B and α-glucosidase, both of which are important targets to treat obesity and diabetes. The principal components were identified as geranylated flavonoids (**1–8**), which showed mixed type inhibition against PTP1B with IC_50_s = 1.9–8.2 μM and noncompetitive inhibition towards α-glucosidase with IC_50_s = 2.2–78.9 μM. Furthermore, detailed inhibitory behaviours were inferred from double-reciprocal plots and resulting kinetic parameters: *V*_max_, *K*_m_, *K*_ik_, and *K*_iv_. This result indicates that geranylated flavonoids, the most effective constituents of this plant, simultaneously inhibit α-glucosidase and PTP1B enzymes. Such compounds may display synergistic effects to antagonize hyperglycaemia, and hence significantly improve insulin sensitization. Further studies using *in vivo* diabetic models are necessary to well define the underlying potential of these interesting compounds.

## Supplementary Material

Supplementary materials

## References

[1] Yip SC, Saha S, Chernoff J. PTP1B: a double agent in metabolism and oncogenesis. Trends Biochem Sci 2010;35:442–9. 20381358 10.1016/j.tibs.2010.03.004 PMC2917533

[2] Bence KK, Delibegovic M, Xue B, et al Neuronal PTP1B regulates body weight, adiposity and leptin action. Nat Med 2006;12:917–24. 16845389 10.1038/nm1435

[3] Bialy L, Waldmann H. Inhibitors of protein tyrosine phosphatases: next-generation drugs? Angew Chem Int Ed Engl 2005;44:3814–39. 15900534 10.1002/anie.200461517

[4] Johnson TO, Ermolieff J, Jirousek MR. Protein tyrosine phosphatase 1B inhibitors for diabetes. Nat Rev Drug Discov 2002;1:696–709. 12209150 10.1038/nrd895

[5] Klaman LD, Boss O, Peroni OD, et al Increased energy expenditure, decreased adiposity, and tissue-specific insulin sensitivity in protein-tyrosine phosphatase 1B-deficient mice. Mol Cell Biol 2000;20:5479–89. 10891488 10.1128/mcb.20.15.5479-5489.2000 PMC85999

[6] Montalibet J, Kennedy BP. Therapeutic strategies for targeting PTP1B in diabetes. Drug Discov Today 2005;2:129–35.

[7] Bojarova P, Kren V. Glycosidases: a key to tailored carbohydrates. Trends Biotechnol 2009;27:199–209. 19250692 10.1016/j.tibtech.2008.12.003

[8] Dwek RA, Butters TD, Platt FM, Zitzmann N. Targeting glycosylation as a therapeutic approach. Nat Rev Drug Discov 2002;1:65–75. 12119611 10.1038/nrd708

[9] Asano N. Glycosidase inhibitors: update and perspectives on practical use. Glycobiology 2003;13:93–104. 10.1093/glycob/cwg090 12851286

[10] Smejkal K, Grycova L, Marek R, et al *C*-Geranyl compounds from *Paulownia tomentosa* fruits. J Nat Prod 2007;70:1244–8. 17625893 10.1021/np070063w

[11] Schneiderova K, Smejkal K. Phytochemical profile of *Paulownia tomentosa* (Thunb). Steud. Phytochem Rev 2015;14:799–833. 10.1007/s11101-014-9376-y PMC7089068 32214918

[12] Hanakova H, Hosek J, Babula P, et al C-geranylated flavanones from *Paulownia tomentosa* fruits as potential anti-inflammatory compounds acting via inhibition of TNF-α production. J Nat Prod 2015;78:850–63. 25735399 10.1021/acs.jnatprod.5b00005

[13] Cho JK, Ryu YB, Curtis-Long MJ, et al Cholinestrase inhibitory effects of geranylated flavonoids from *Paulownia tomentosa* fruits. Bioorg Med Chem 2012;20:2595–602. 22445674 10.1016/j.bmc.2012.02.044

[14] Zima A, Hosek J, Treml J, et al Antiradical and cytoprotective activities of several C-geranyl-substituted flavanones from *Paulownia tomentosa* fruit. Molecules 2010;15:6035–49. 20877208 10.3390/molecules15096035 PMC6257673

[15] Lee Y, Ryu YB, Youn HS, et al Structural basis of sialidase in complex with geranylated flavonoids as potent natural inhibitors. Acta Crystallogr D Biol Crystallogr 2014;D70:1357–65. 10.1107/S1399004714002971 PMC4014123 24816104

[16] Asai T, Hara N, Kobayashi S, et al Geranylated flavanones from the secretion on the surface of the immature fruits of *Paulownia tomentosa*. Phytochemistry 2008;69:1234–41. 18206191 10.1016/j.phytochem.2007.11.011

[17] Smejkal K, Babula P, Slapetova T, et al Cytotoxic activity of *C*-Geranyl compounds from *Paulownia tomentosa* fruits. Planta Med 2008;74:1488–91. 18729043 10.1055/s-2008-1081339

[18] Fang L, Cao J, Duan L, et al Protein tyrosine phosphatase 1B (PTP1B) and α-glucosidase inhibitory activities of *Schisandra chinensis* (Turcz.) Baill. J Funct Foods 2014;9:264–70.

[19] Ryu HW, Cho JK, Curtis-Long MJ, et al α-Glucosidase inhibition and antihyperglycemic activity of prenylated xanthones from *Garcinia mangostana*. Phytochemistry 2011;72:2148–54. 21872893 10.1016/j.phytochem.2011.08.007

[20] Chiari ME, Vera DMA, Palacios SM, Carpinella MC. Tyrosinase inhibitory activity of a 6-isoprenoid-substituted flavanone isolated from Dalea elegans. Bioorg Med Chem 2011;19:3474–82. 21561780 10.1016/j.bmc.2011.04.025

[21] Yang X, Du Z, Pu J, et al Classification of difference between inhibition constants of an inhibitor to facilitate identifying the inhibition type. J Enzyme Inhib Med Chem 2013;28:205–13. 22224402 10.3109/14756366.2011.645240

[22] Adisakwattana S, Ngamrojanavanich N, Kalampakorn K, et al Inhibitory Activity of Cyanidin-3-rutinoside on alpha-glucosidase. J Enzyme Inhib Med Chem 2004;19:313–16. 15558946 10.1080/14756360409162443

[23] Jung HA, Paudel P, Seong SH, et al Structure related protein tyrosine phosphatase 1B inhibition by naringenin derivatives. Bioorg Med Chem Lett 2017;27:2274–80. 28454670 10.1016/j.bmcl.2017.04.054

[24] Uddin Z, Song YH, Curtis-Long MJ, et al Potent bacterial neuraminidase inhibitors, anthraquinone glucosides from *Polygonum cuspidatum* and their inhibitory mechanism. J Ethnopharmacol 2016;193:283–92. 27553976 10.1016/j.jep.2016.08.026

[25] Xie F, Gong S, Zhang W, et al Potential of lignin from *Canna edulis* ker residue in the inhibition of α-D-glucosidase: Kinetics and interaction mechanism merging with docking simulation. Int J Biol Macromol 2017;95:592–602. 27908712 10.1016/j.ijbiomac.2016.11.100

