# Supplementary material for: Inhibition of protein tyrosine phosphatase (PTP1B) and α-glucosidase by geranylated flavonoids from Paulownia tomentosa
Source: J Enzyme Inhib Med Chem. 2017 Sep 21;32(1):1195–202. doi: 10.1080/14756366.2017.1368502 (PMC6010085; doi:10.1080/14756366.2017.1368502)
Supplement: Supplementary materials [file IENZ_A_1368502_SM1086.pdf]

SUPPLEMENTARY MATERIALS

Inhibition of protein tyrosine phosphatase (PTP1B) and α-glucosidase by geranylated flavonoids from *Paulownia tomentosa*

Yeong Hun Song[a,#], Zia Uddin[a,#], Young Min Jin[a], Zuopeng Li[a], Marcus John Curtis-Long[b], Kwang Dong Kim[c], Jung Keun Cho[a] and Ki Hun Park[a,]*

[a] *Division of Applied Life Science (BK21 plus), Institute of Agriculture and Life Science (IALS), Gyeongsang National University, Jinju 52828, Republic of Korea:* [b] *Department of Chemistry and Chemical Biology, Cornell University, Ithaca, New York 14853, United States:* [c] *Division of Applied Life Science (BK21 plus), PMBBRC, Gyeongsang National University, Jinju 52828, Republic of Korea*

▣ Characterization Data

S2-4 : NMR and HREIMS data of compound **1**

S5-7 : NMR and HREIMS data of compound **8**

S8-19 : NMR and HREIMS data of compounds **2-7**

S20-23 : Isolated compound NMR assign data (^{1}H, ^{13}C)

S24 : CD spectrum of compounds **1-8**

S25-26 : Enzyme kinetic data of PTP1B

S27-28 : Enzyme kinetic data of α-glucosidase

S29 : Mixed type parameter of PTP1B (K_I,K_{IS})

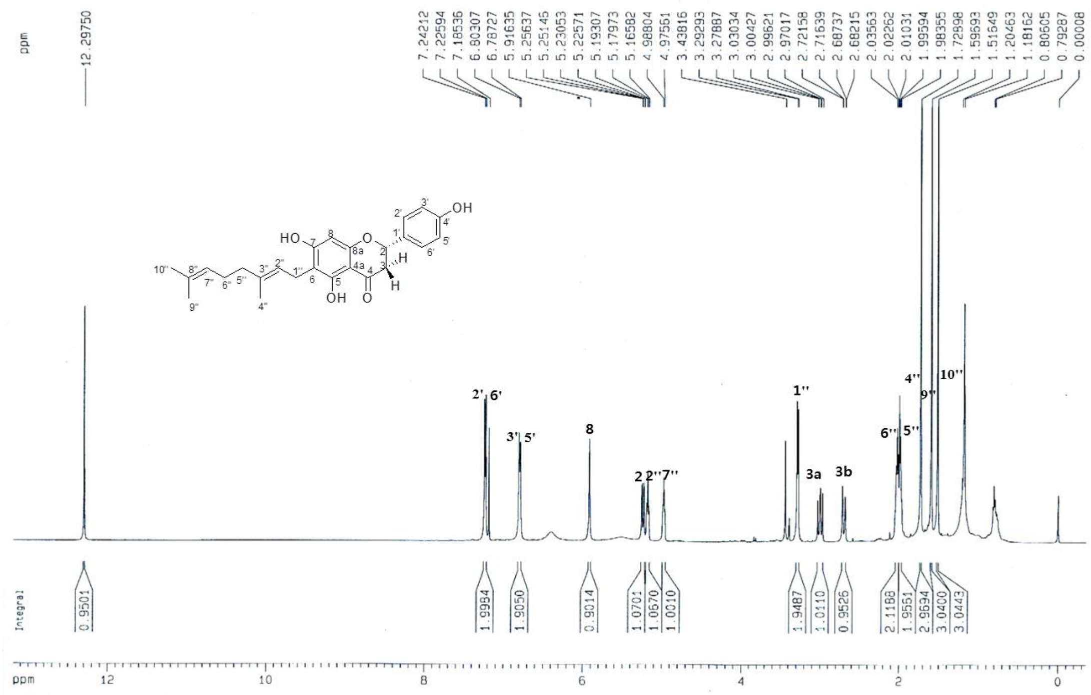

Figure 1. ^{1}H-NMR spectrum of compound **1** (500MHz, $CDCl_3$).

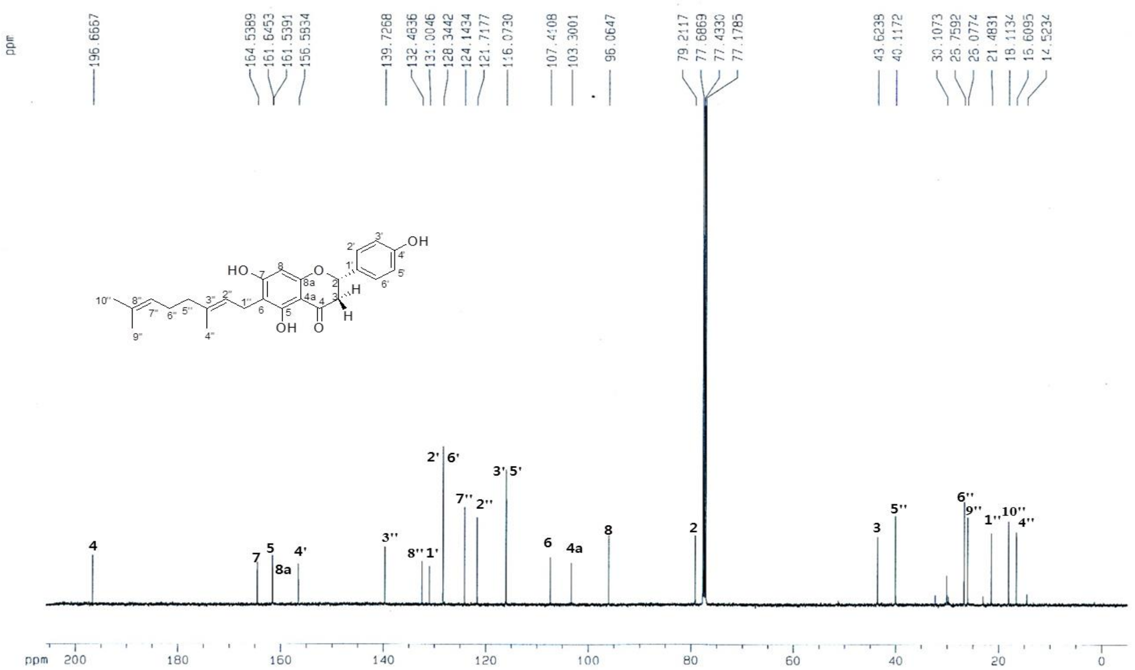

Figure 2. ^{13}C-NMR spectrum of compound **1** (125MHz, $CDCl_3$).

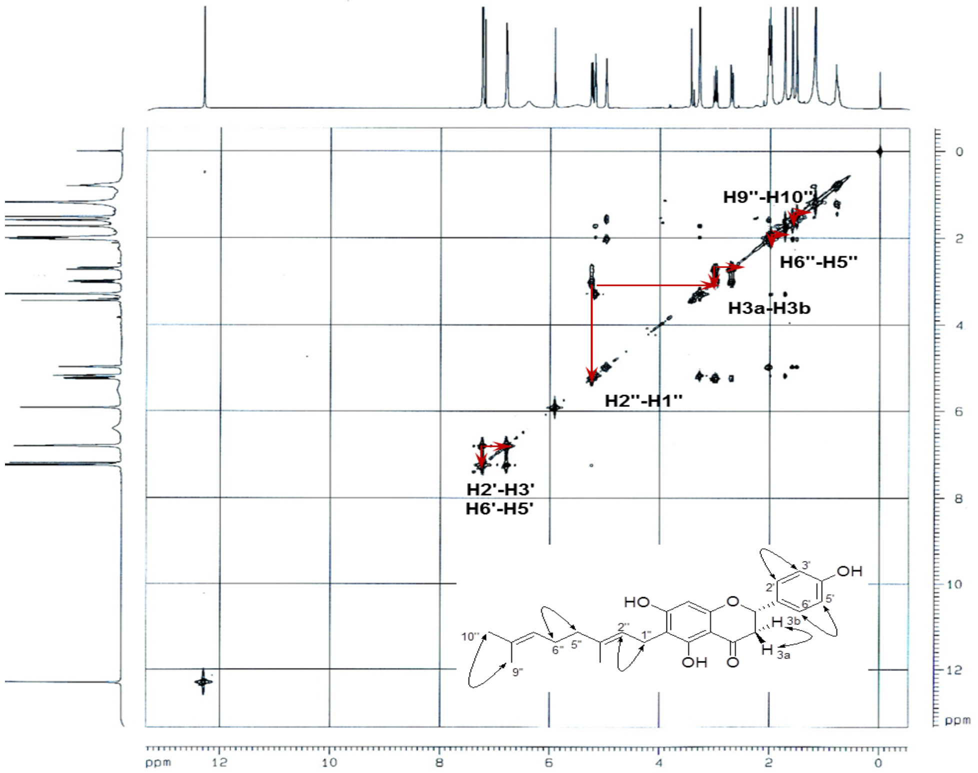

Figure 3. ^1H-^1H COSY spectrum of compound **1**.

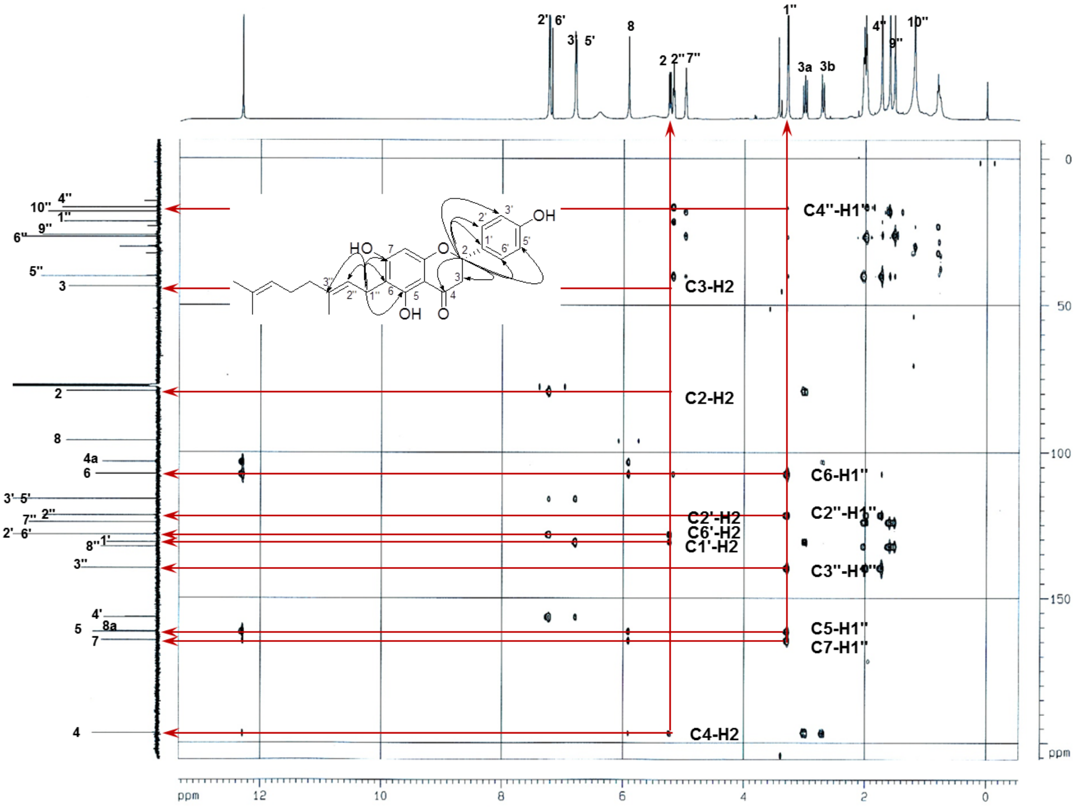

Figure 4. HMBC spectrum of compound **1**.

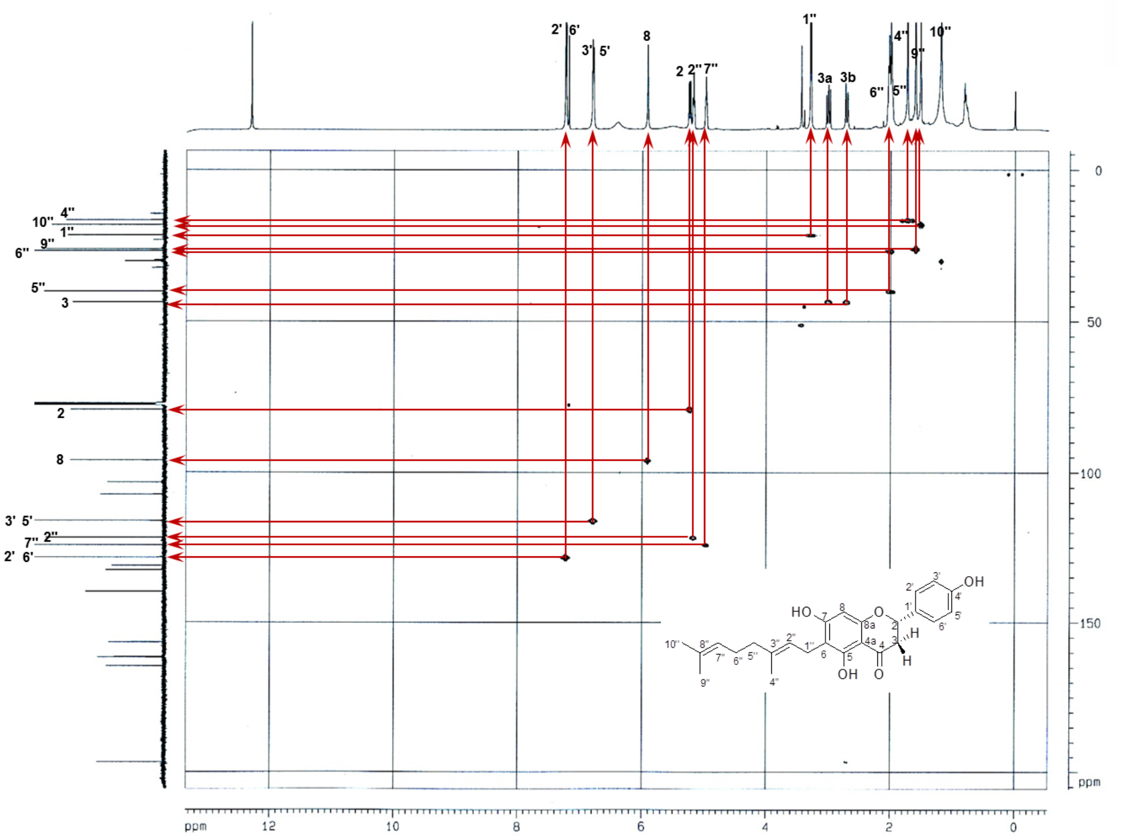

Figure 5. HMQC spectrum of compound **1**.

```
[ Elemental Composition ]
Data   :
Sample: -
Note  : -
Inlet  : Direct                                Ion Mode : EI+
RT : 1.95 min                                  Scan#: 40
Elements : C 100/1, H 100/0, O 20/1
Mass Tolerance         : 1000ppm, 3mmu if m/z < 3, 5mmu if m/z > 5
Unsaturation (U.S.) : 3.0 - 30.0

Observed m/z  Int%    Err[ppm / mmu]    U.S.  Composition
  408.1938    100.0      +0.1 /  +0.0    3.0  C 25 H 28 O 5
```

Figure 6. HREIMS data of compound **1**.

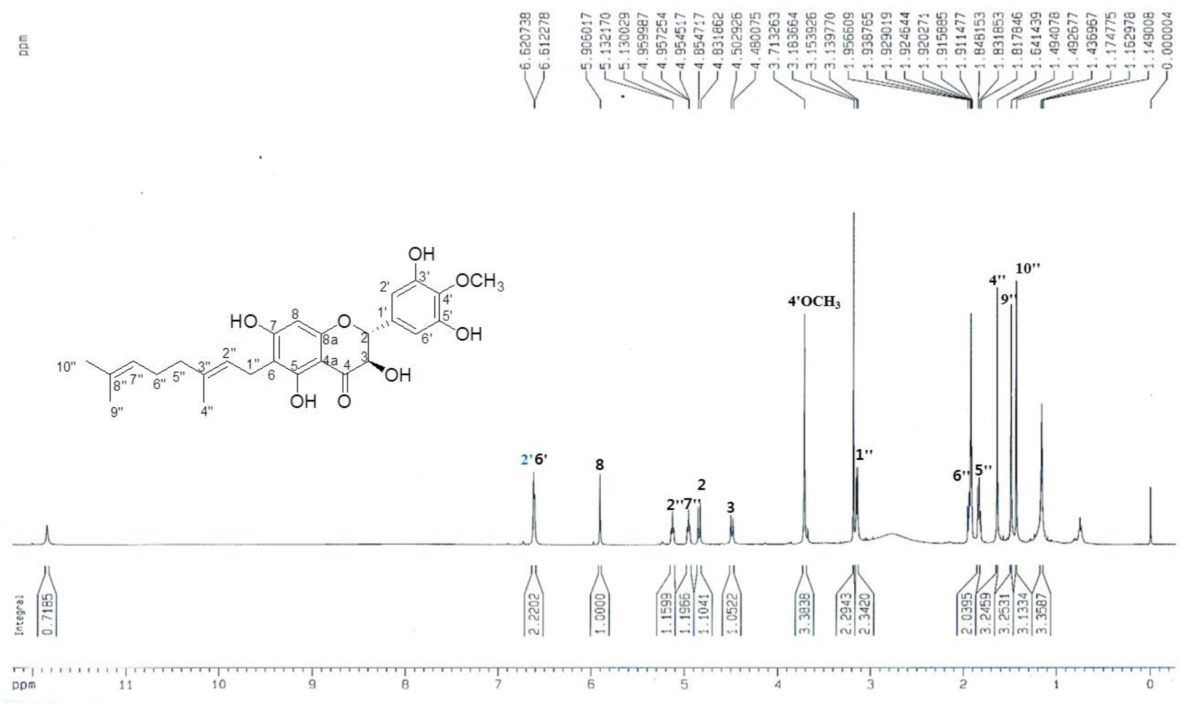

Figure 7. ^{1}H-NMR spectrum of compound **8** (500MHz, Aceton-d_6).

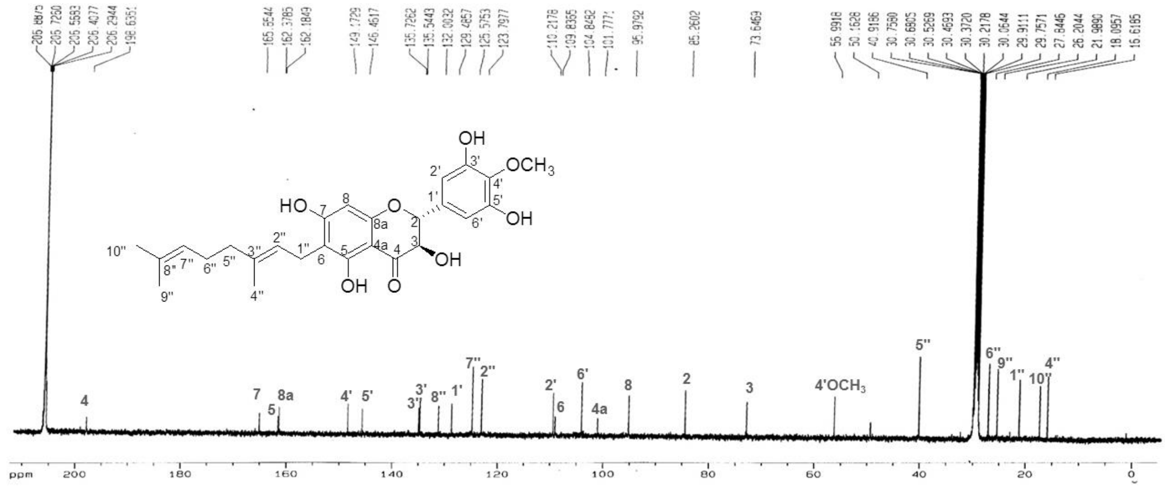

Figure 8. ^{13}C-NMR spectrum of compound **8** (125MHz, Aceton-d_6).

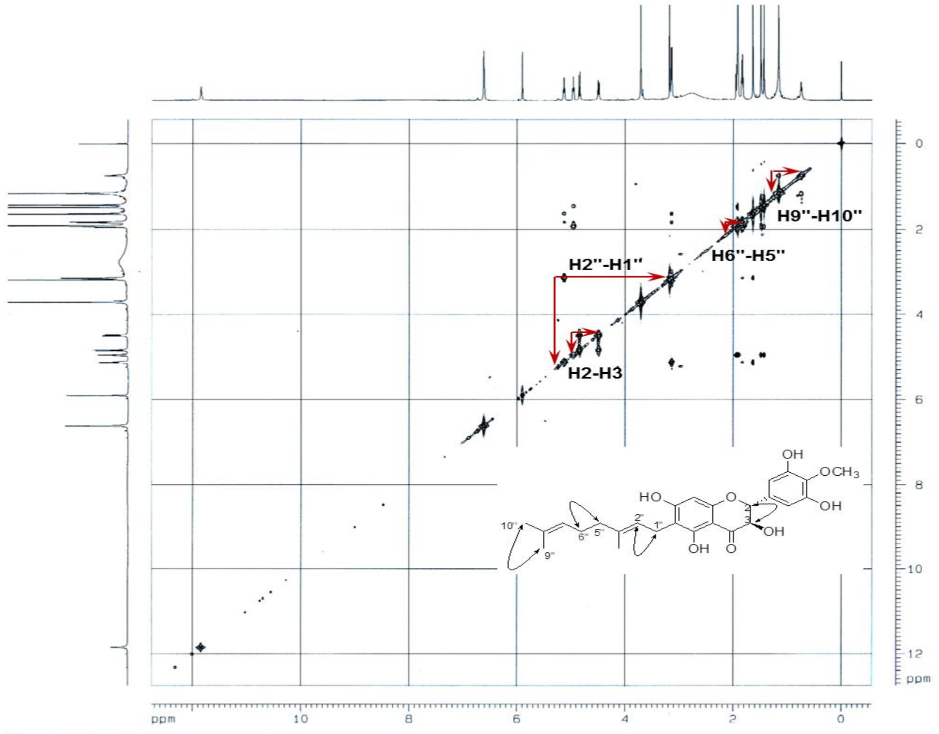

Figure 9. ^{1}H-^{1}H COSY spectrum of compound **8**.

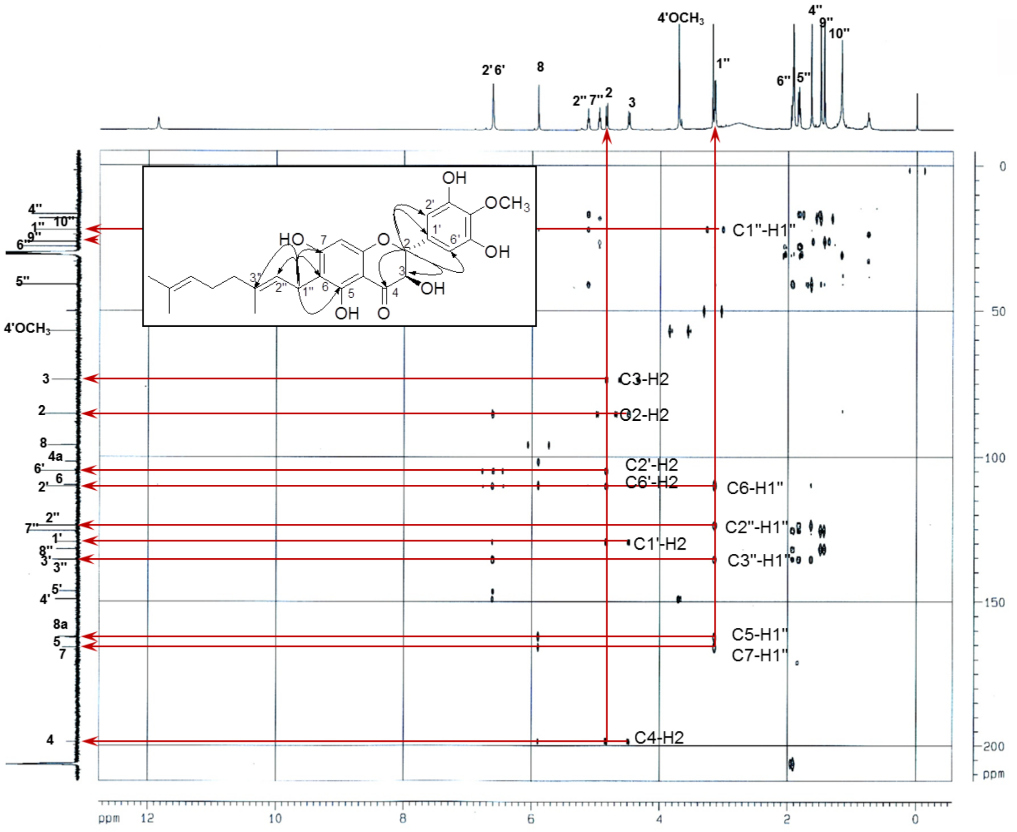

Figure 10. HMBC spectrum of compound **8**.

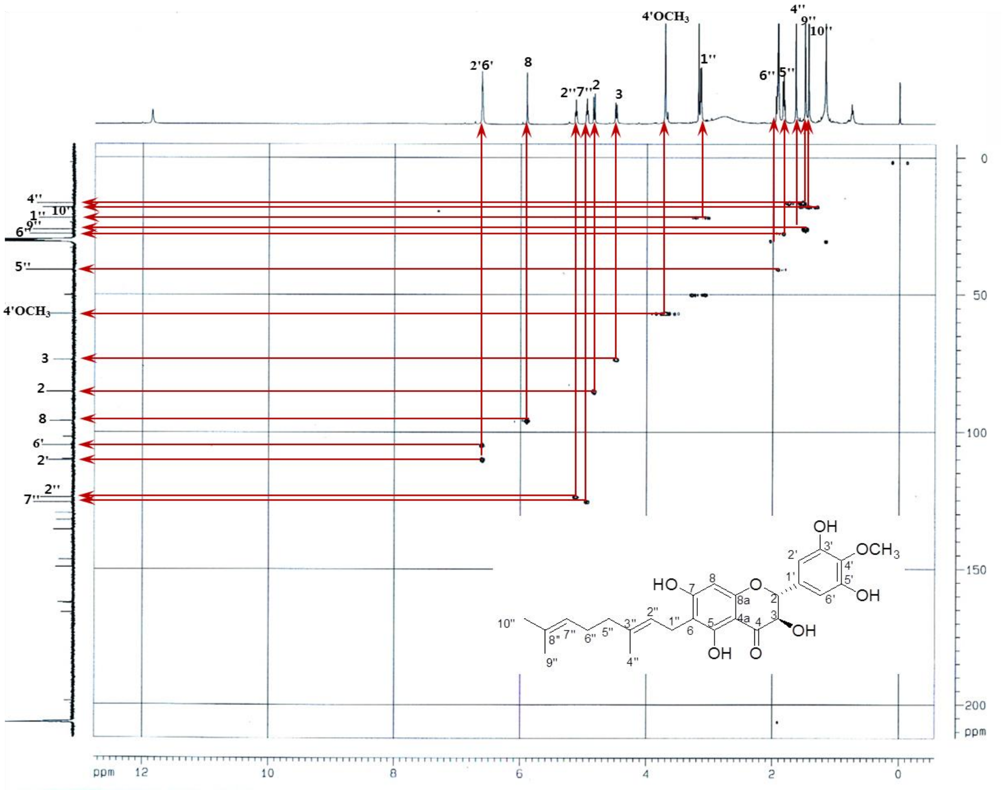

Figure 11. HMQC spectrum of compound **8**.

```
[ Elemental Composition ]
Data   :
Sample: -
Note : -
Inlet : Direct                           Ion Mode : EI+
RT : 2.20 min                            Scan#: 45
Elements : C 100/1, H 100/1, O 20/1
Mass Tolerance       : 1000ppm, 1mmu if m/z < 1, 10mmu if m/z > 10
Unsaturation (U.S.)  : 1.0 - 30.0

Observed m/z  Int%    Err[ppm / mmu]    U.S.   Composition
  470.1943   100.0     +13.0 /  +6.1    21.0   C 33 H 26 O 3
                        +0.5 /  +0.2    12.0   C 26 H 30 O 8
                       -12.0 /  -5.6     3.0   C 19 H 34 O 13
```

Figure 12. HREIMS data of compound **8**.

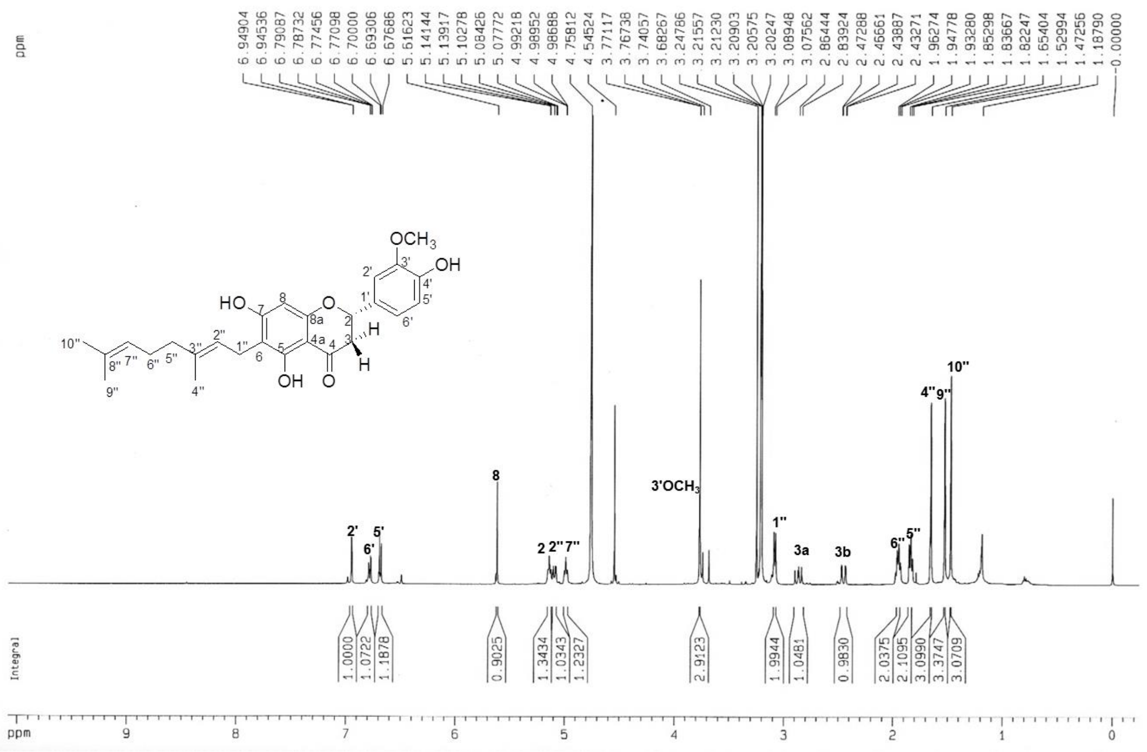

Figure 13. ^{1}H-NMR spectrum of compound **2** (500MHz, CD_3OD).

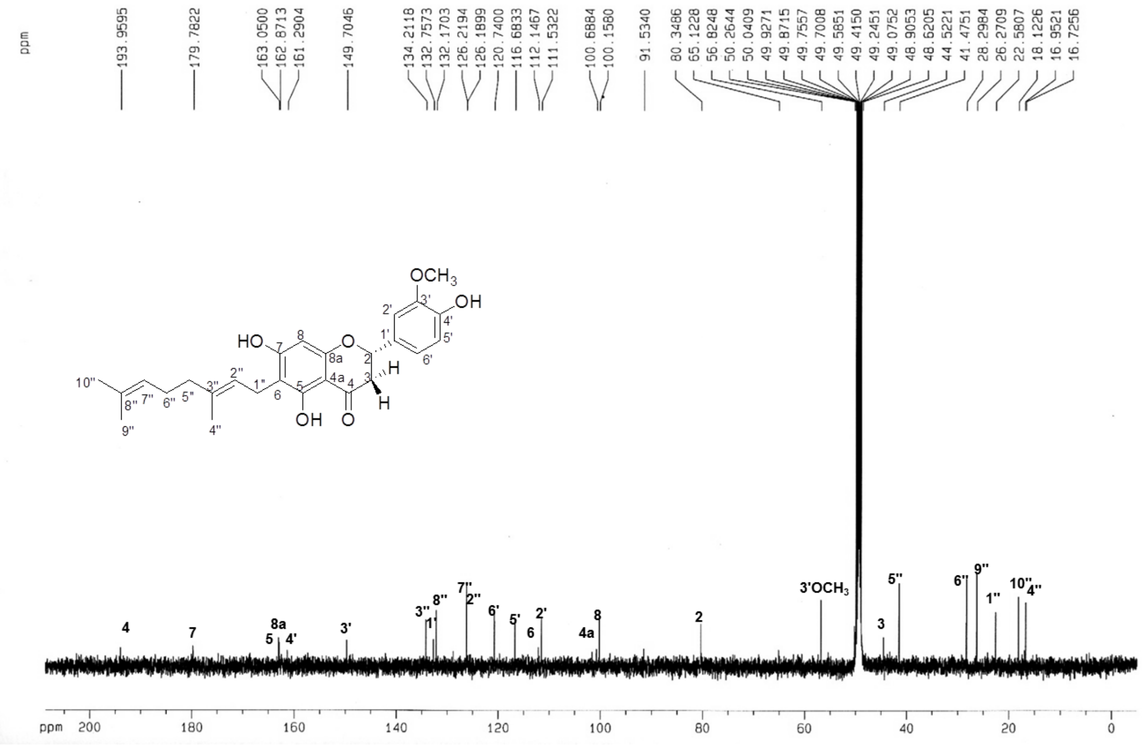

Figure 14. ^{13}C-NMR spectrum of compound **2** (125MHz, CD_3OD).

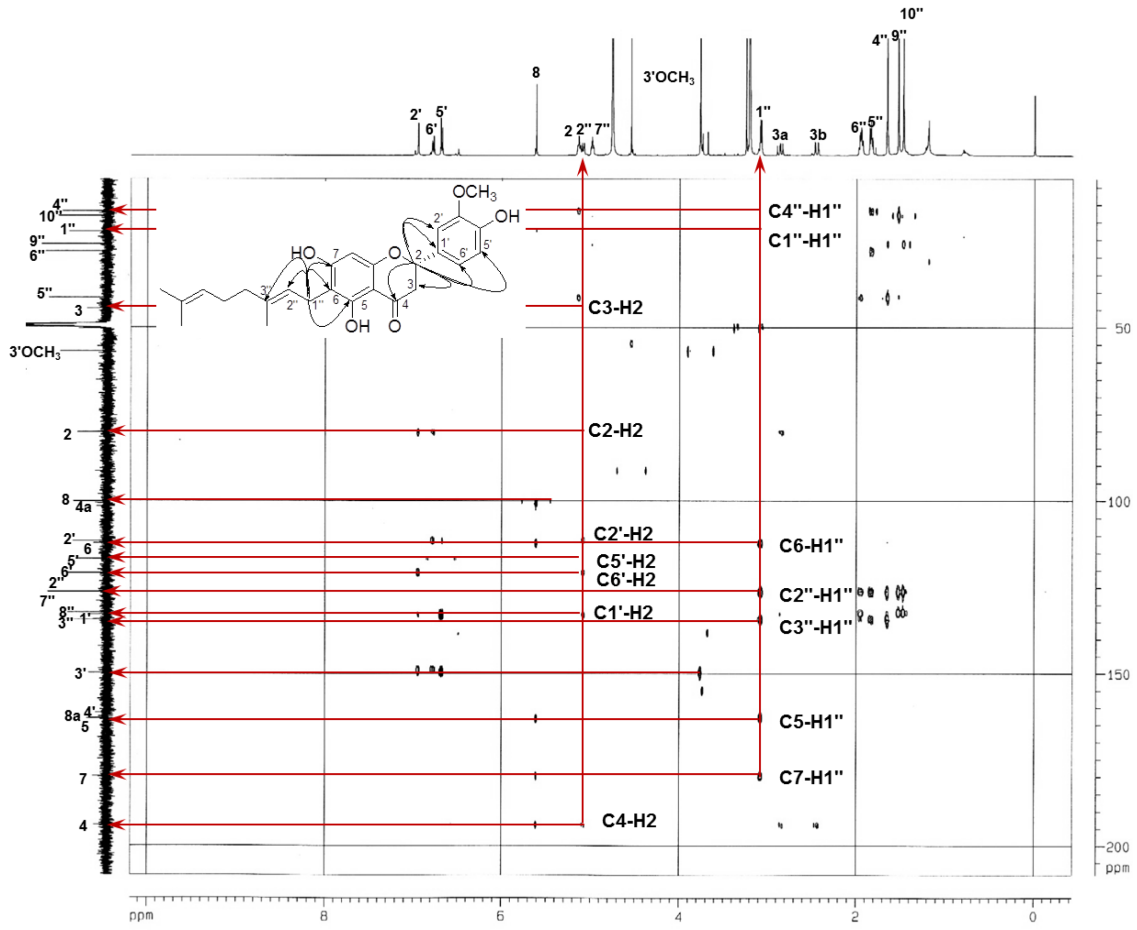

Figure 15. HMBC spectrum of compound **2**.

```
[ Elemental Composition ]
Data   :
Sample: -
Note  : -
Inlet : Direct                              Ion Mode : EI+
RT  : 2.50 min                              Scan#: 76
Elements : C 100/1, H 100/1, O 20/1
Mass Tolerance       : 1000ppm, 1mmu if m/z < 1, 10mmu if m/z > 10
Unsaturation (U.S.)  : 5.0 - 30.0

Observed m/z  Int%     Err[ppm / mmu]     U.S.   Composition
  438.2035   100.0      +11.6 /  +5.1     21.0   C 33 H 26 O
                         -1.8 /  -0.8     12.0   C 26 H 30 O 6
```

Figure 16. HREIMS data of compound **2**.

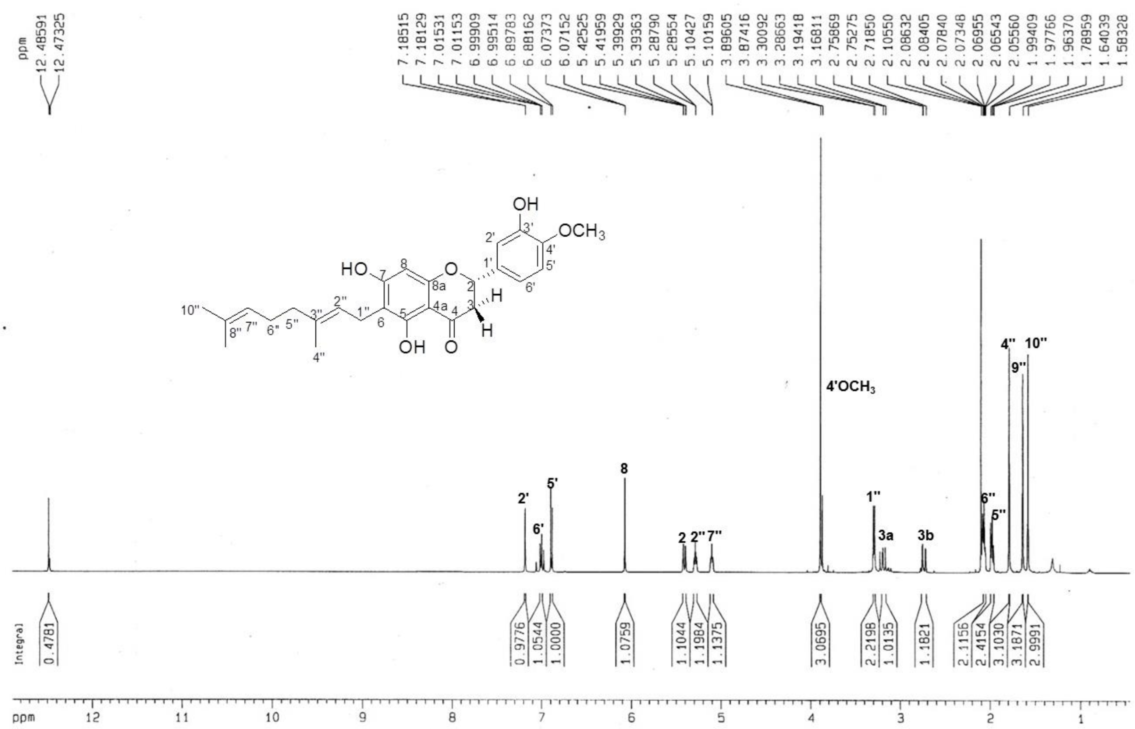

Figure 17. ^{1}H-NMR spectrum of compound **3** (500MHz, Aceton-d_6).

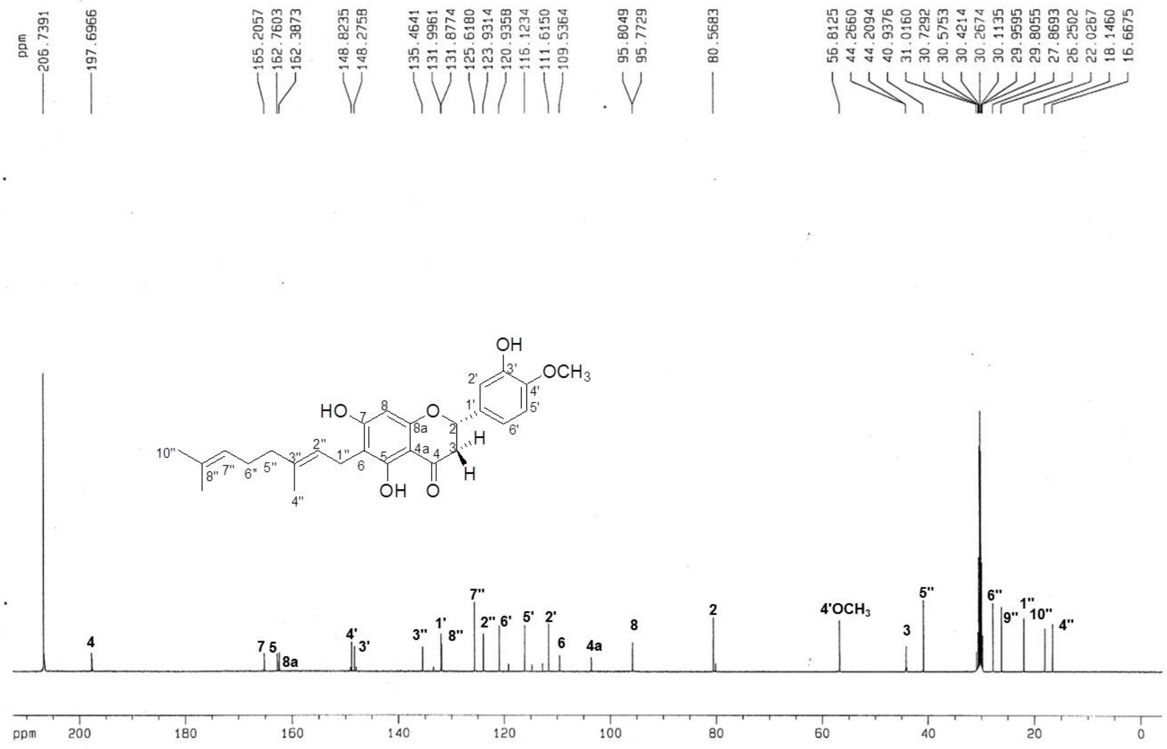

Figure 18. ^{13}C-NMR spectrum of compound **3** (125MHz, Aceton-d_6).

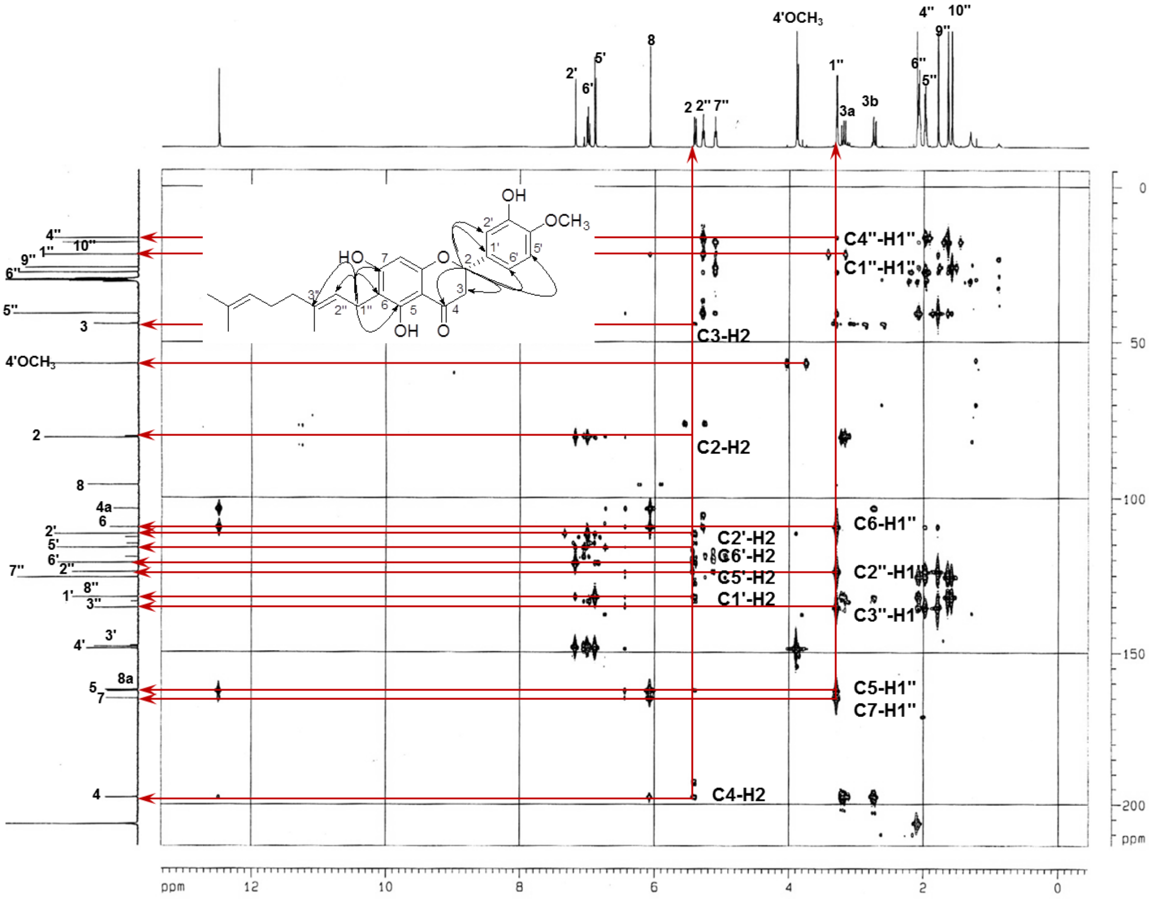

Figure 19. HMBC spectrum of compound **3**.

```
[ Elemental Composition ]
Data   :
Sample: -
Note  : -
Inlet  : Direct                              Ion Mode  : EI+
RT  : 1.90 min                               Scan#: 58
Elements : C 100/1, H 100/1, O 20/1
Mass Tolerance          : 1000ppm, 1mmu if m/z < 1, 10mmu if m/z > 10
Unsaturation (U.S.)  : 5.0 - 30.0

Observed m/z  Int%    Err[ppm / mmu]    U.S.   Composition
  438.2040   100.0     +13.0 /  +5.7    21.0   C 33 H 26 O
                        -0.5 /  -0.2    12.0   C 26 H 30 O 6
```

Figure 20. HREIMS data of compound **3**.

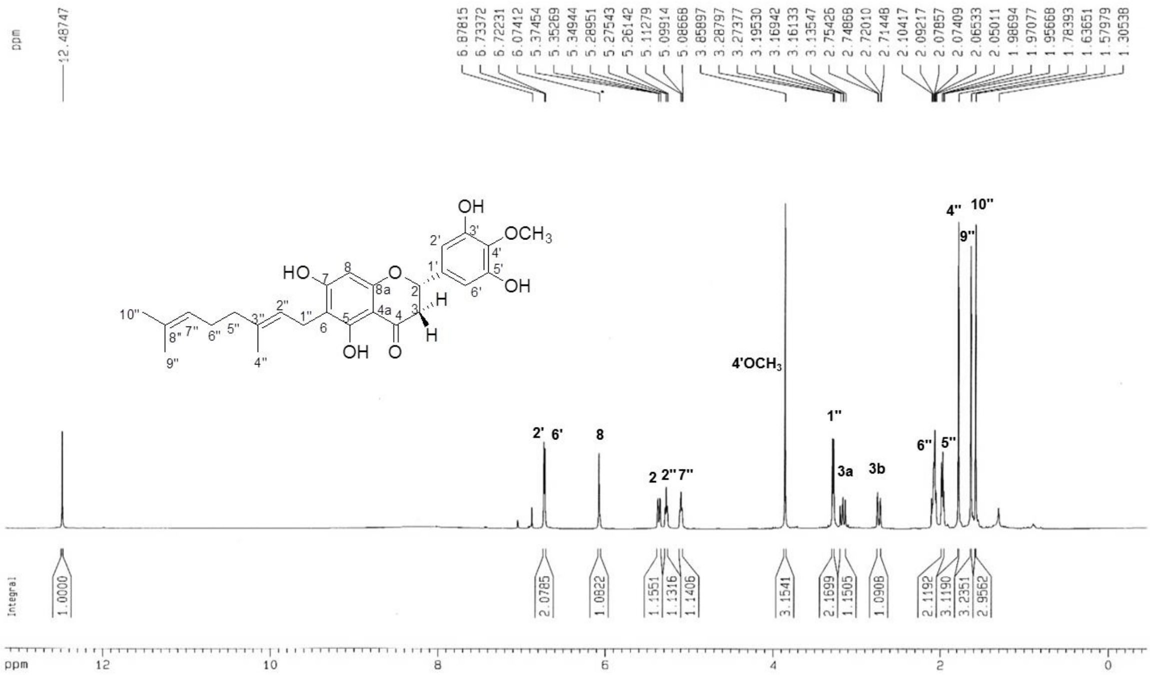

Figure 21. ^{1}H-NMR spectrum of compound **4** (500MHz, Aceton-d_6).

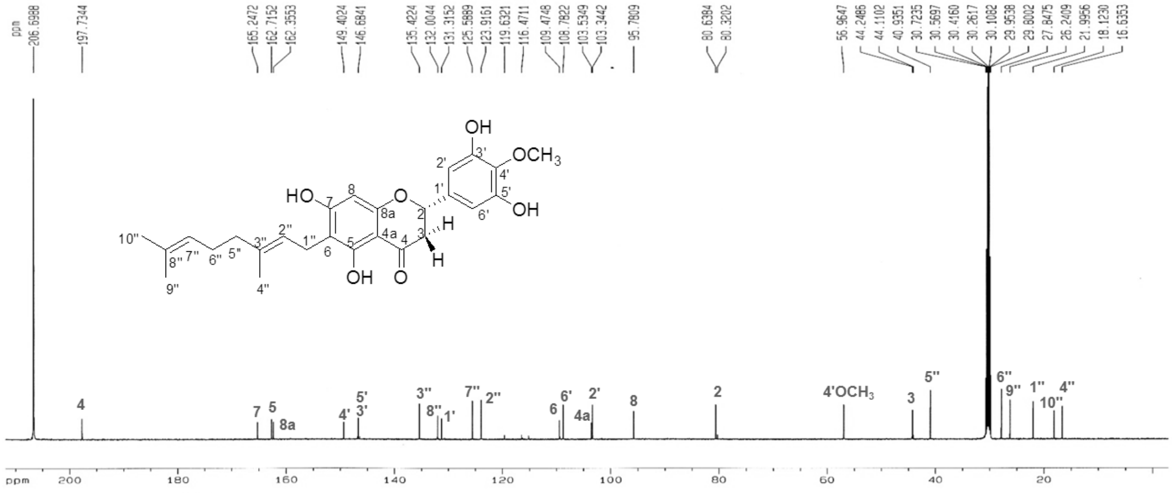

Figure 22. ^{13}C-NMR spectrum of compound **4** (125MHz, Aceton-d_6).

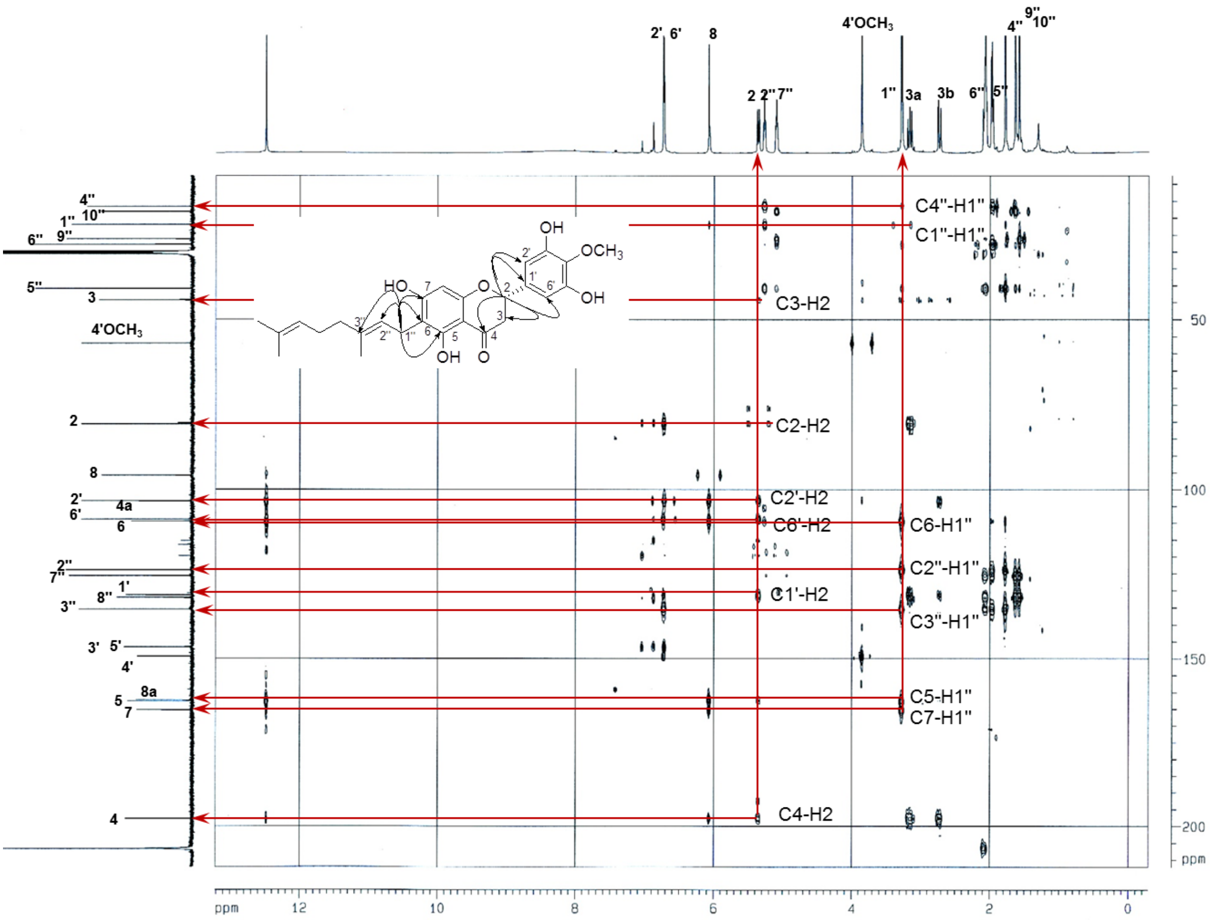

Figure 23. HMBC spectrum of compound **4**.

```
[ Elemental Composition ]
Data   :
Sample: -
Note  : -
Inlet  : Direct                              Ion Mode : EI+
RT : 1.95 min                                Scan#: 40
Elements : C 100/1, H 100/0, O 20/1
Mass Tolerance        : 1000ppm, 3mmu if m/z < 3, 5mmu if m/z > 5
Unsaturation (U.S.)  : 3.0 - 30.0

Observed m/z  Int%   Err[ppm / mmu]    U.S.   Composition
  424.1947    71.8      +0.6 /  +0.2    3.0   C 18 H 32 O 11

  425.1989    27.4      +5.7 /  +2.4   11.5   C 25 H 29 O 6

  429.0897    15.3      -4.3 /  -1.8   26.5   C 32 H 13 O 2
                        +3.9 /  +1.7    4.5   C 14 H 21 O 15

  454.1992   100.0      +0.1 /  +0.0   12.0   C 26 H 30 O 7
```

Figure 24. HREIMS data of compound **4**.

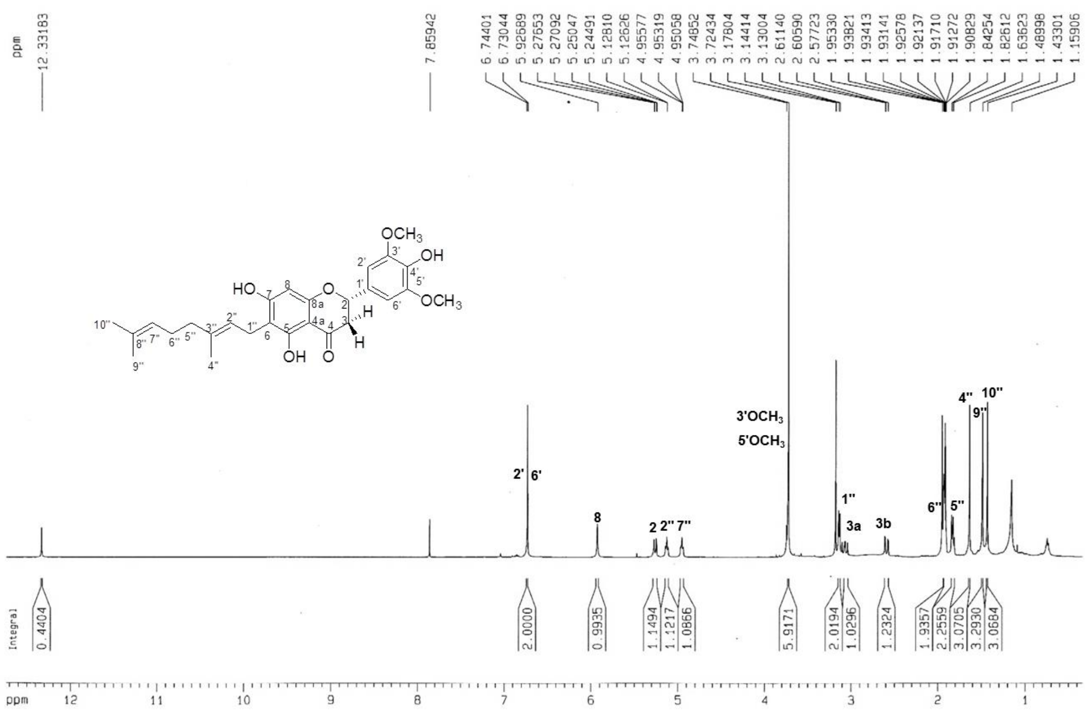

Figure 25. ^{1}H-NMR spectrum of compound **5** (500MHz, Aceton-d_6).

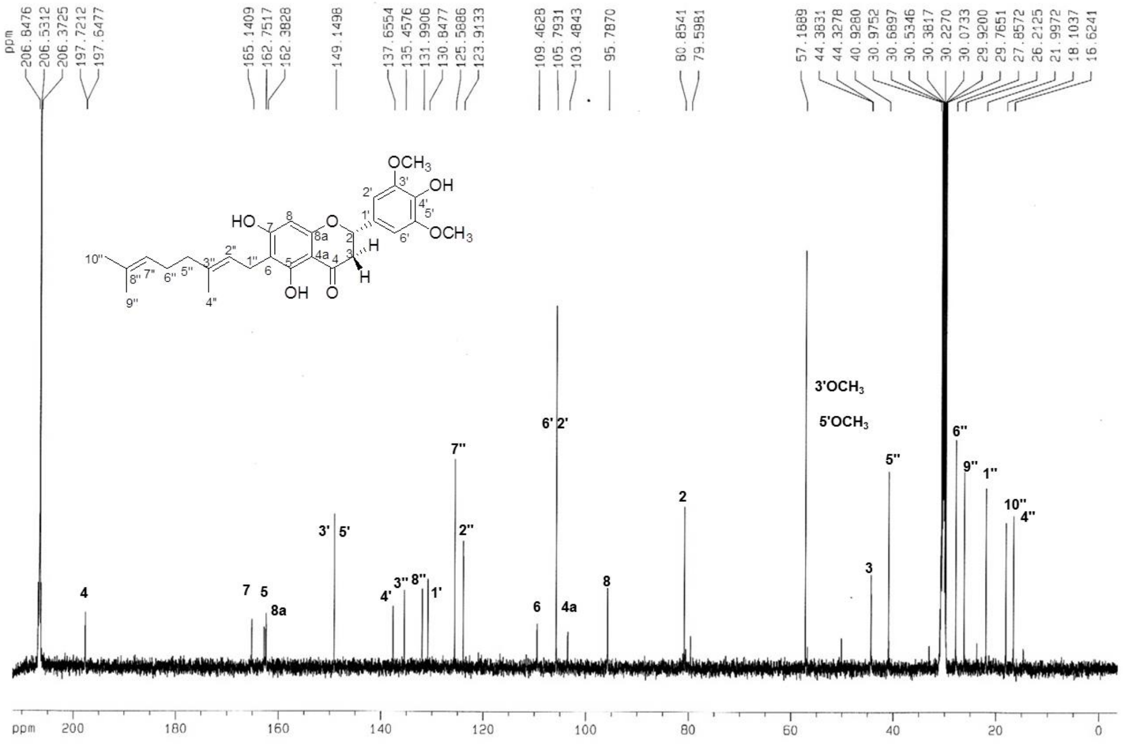

Figure 26. ^{13}C-NMR spectrum of compound **5** (125MHz, Aceton-d_6).

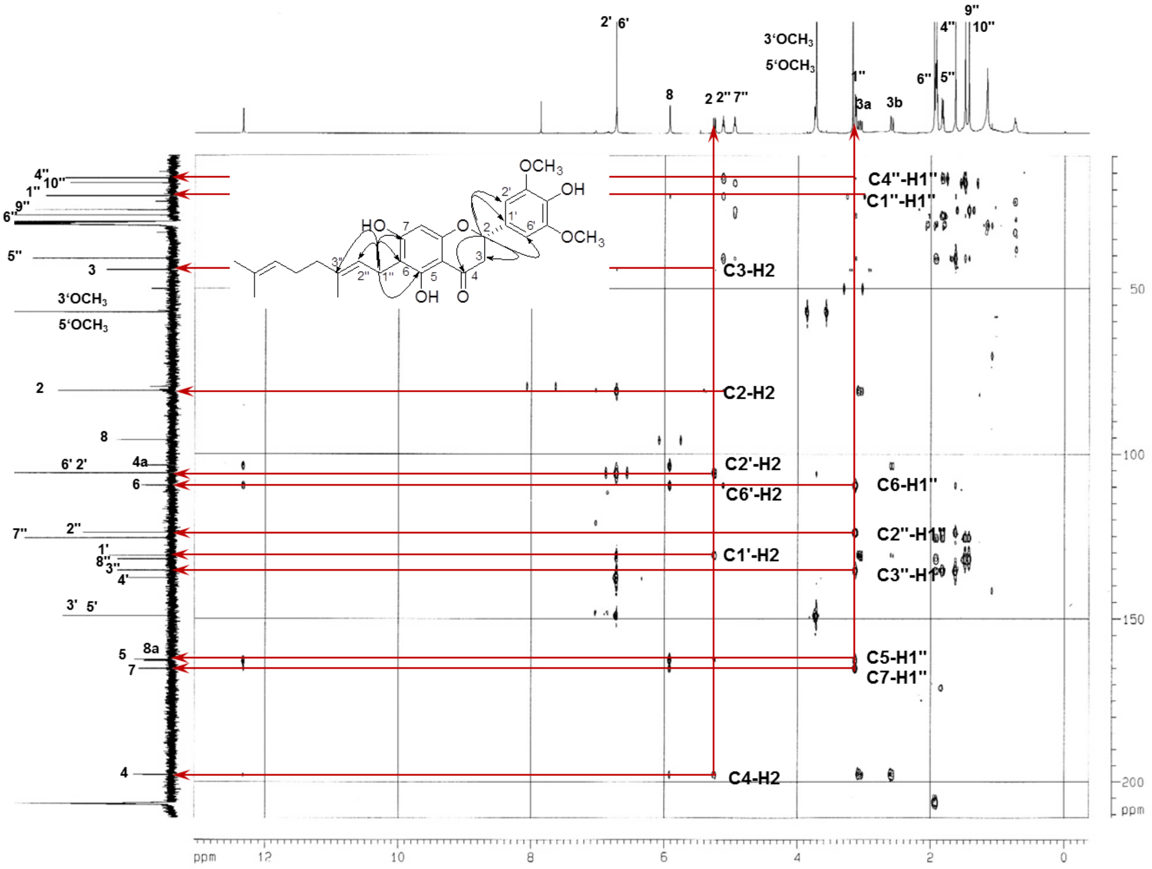

Figure 27. HMBC spectrum of compound **5**.

[Elemental Composition]
Data :
Sample: -
Note : -
Inlet : Direct   Ion Mode : EI+
RT : 1.30 min   Scan#: 40
Elements : C 100/1, H 100/1, O 20/1
Mass Tolerance : 1000ppm, 1mmu if m/z < 1, 10mmu if m/z > 10
Unsaturation (U.S.) : 5.0 - 30.0

Observed m/z	Int%	Err[ppm / mmu]	U.S.	Composition
468.2151	100.0	+13.3 / +6.2	21.0	C 34 H 28 O 2
		+0.7 / +0.3	12.0	C 27 H 32 O 7

Figure 28. HREIMS data of compound **5**.

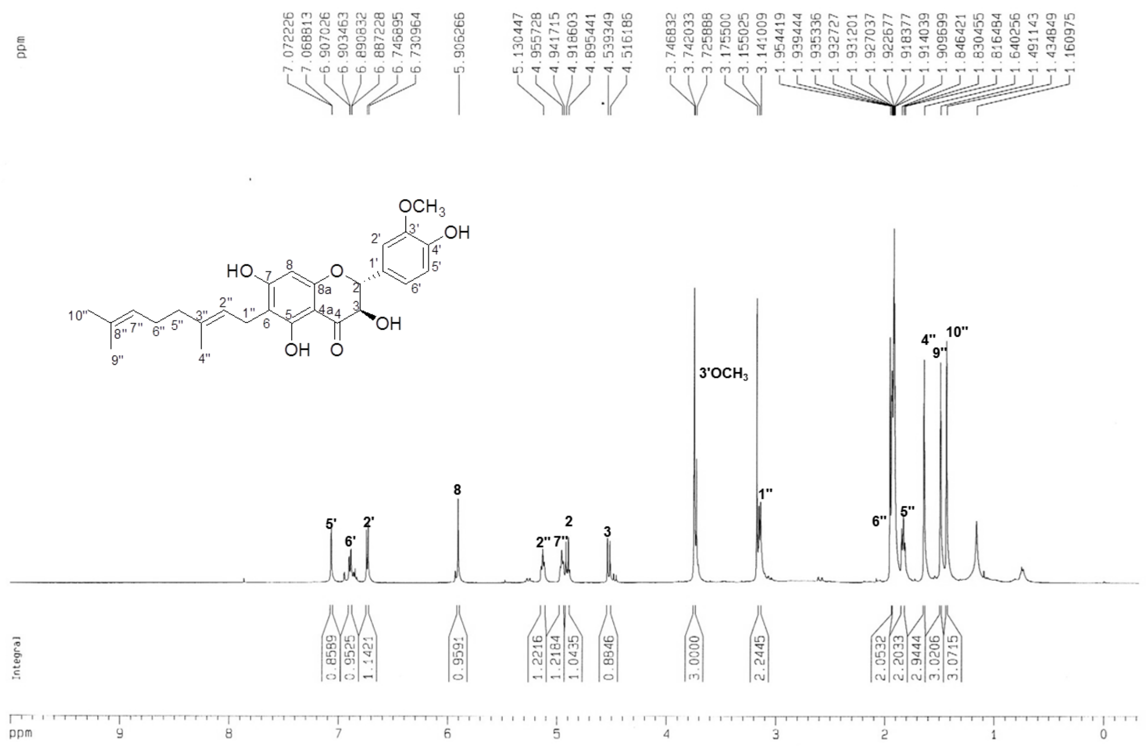

Figure 29. ^{1}H-NMR spectrum of compound **6** (500MHz, Aceton-d_6).

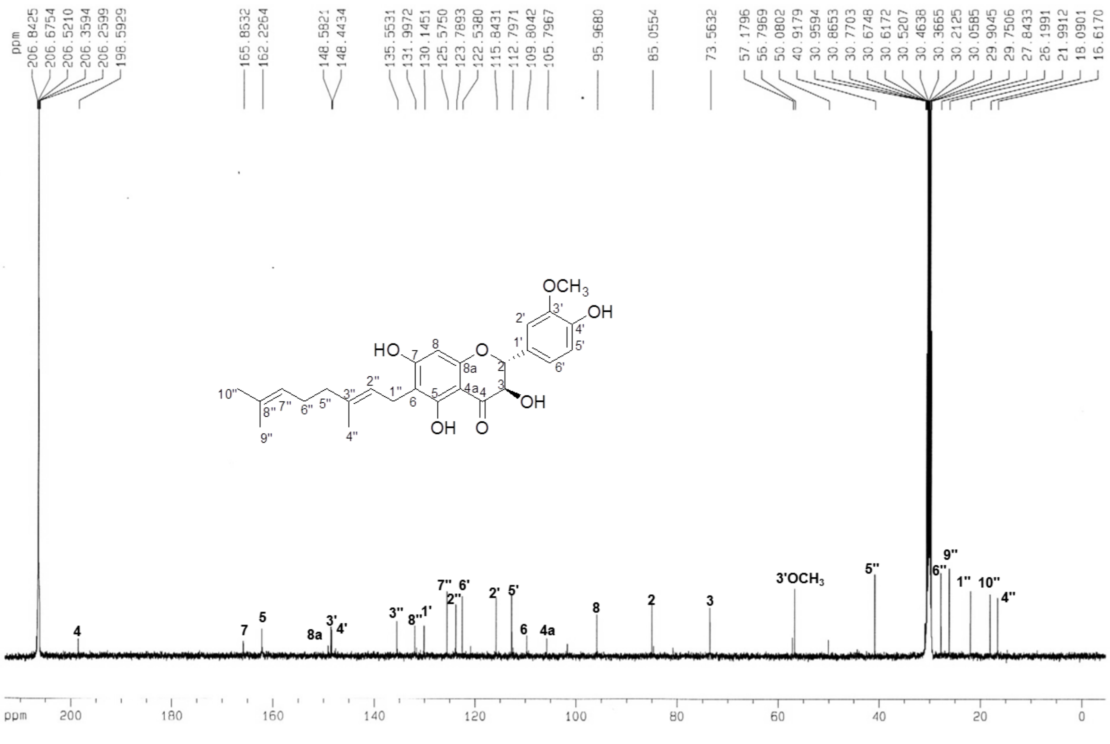

Figure 30. ^{13}C-NMR spectrum of compound **6** (125MHz, Aceton-d_6).

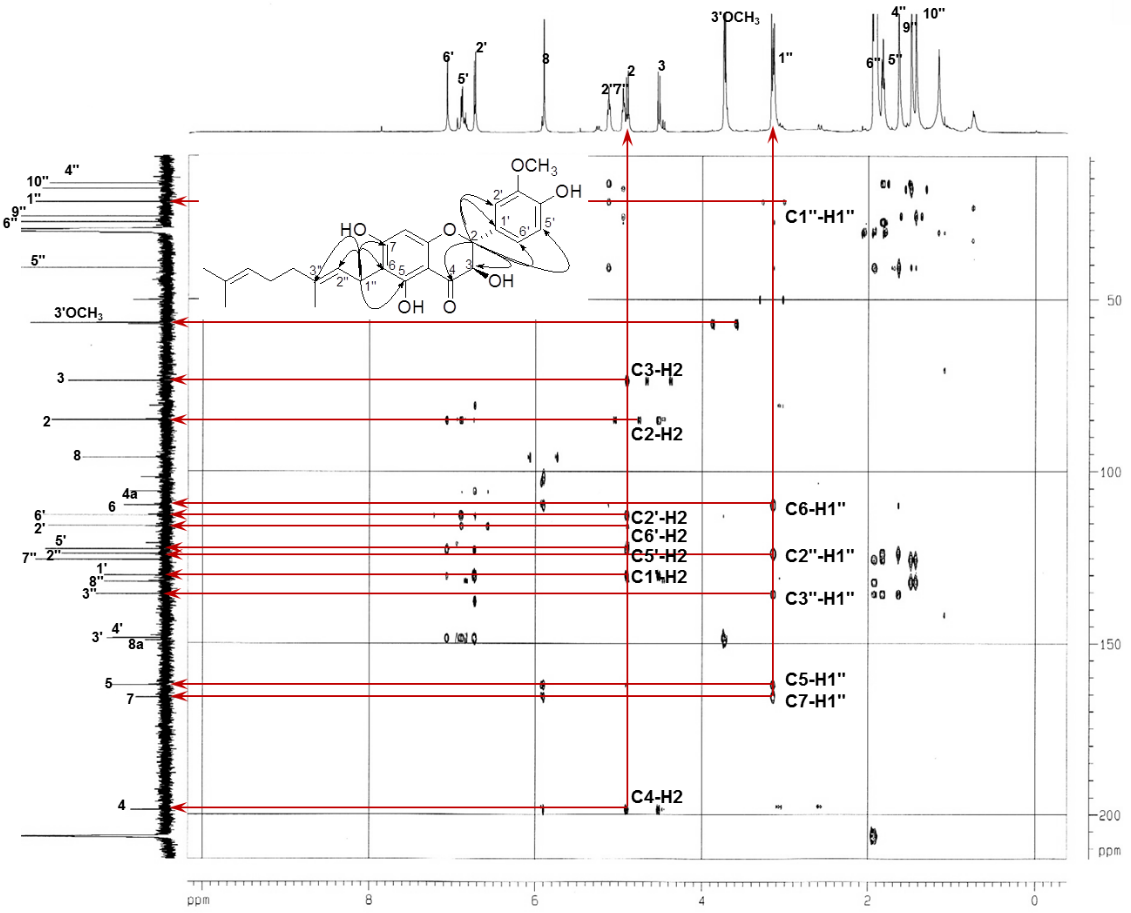

Figure 31. HMBC spectrum of compound **6**.

```
[ Elemental Composition ]
Data   :
Sample: -
Note  : -
Inlet  : Direct                              Ion Mode : EI+
RT : 2.00 min                                Scan#: 41
Elements : C 100/1, H 100/0, O 20/1
Mass Tolerance       : 1000ppm, 3mmu if m/z < 3, 10mmu if m/z > 10
Unsaturation (U.S.) : 3.0 - 30.0

Observed m/z  Int%    Err[ppm / mmu]     U.S.   Composition
  454.1990    100.0    +12.6 /  +5.7     21.0   C 33 H 26 O 2
                        -0.3 /  -0.1     12.0   C 26 H 30 O 7
                       -13.3 /  -6.0      3.0   C 19 H 34 O 12
```

Figure 32. HREIMS data of compound **6**.

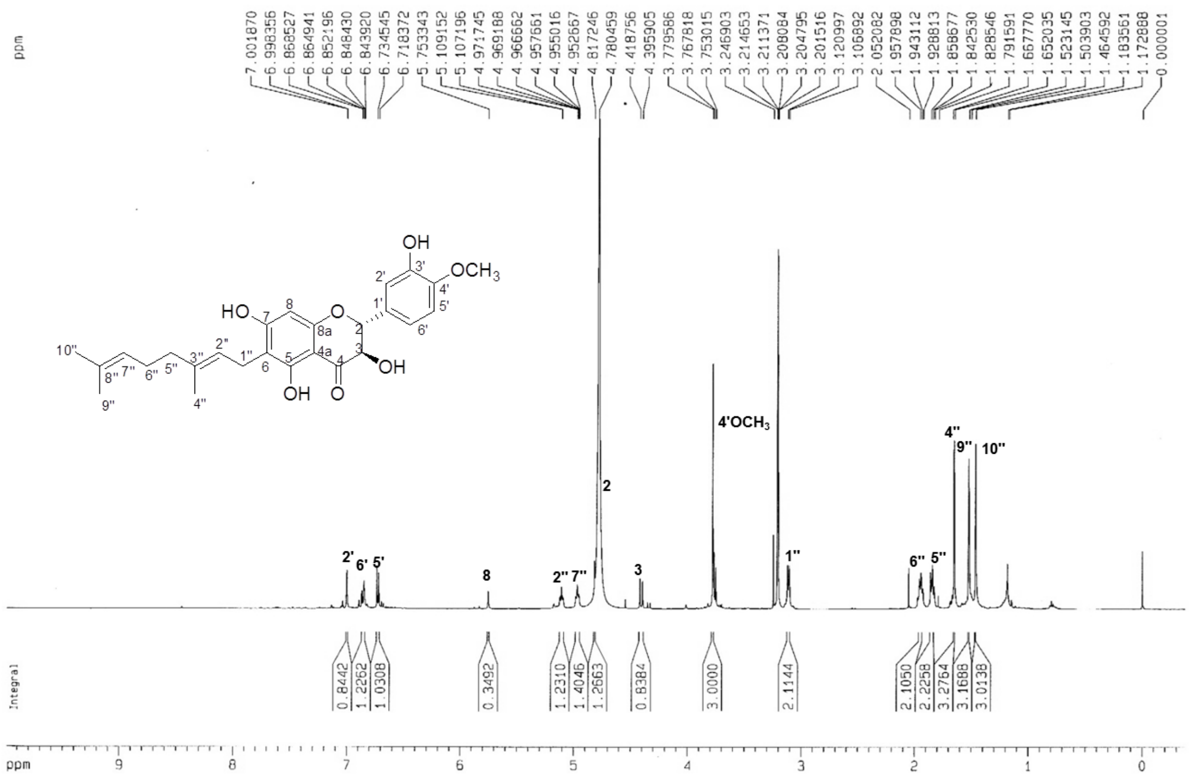

Figure 33. ^{1}H-NMR spectrum of compound **7** (500MHz, CD_3OD).

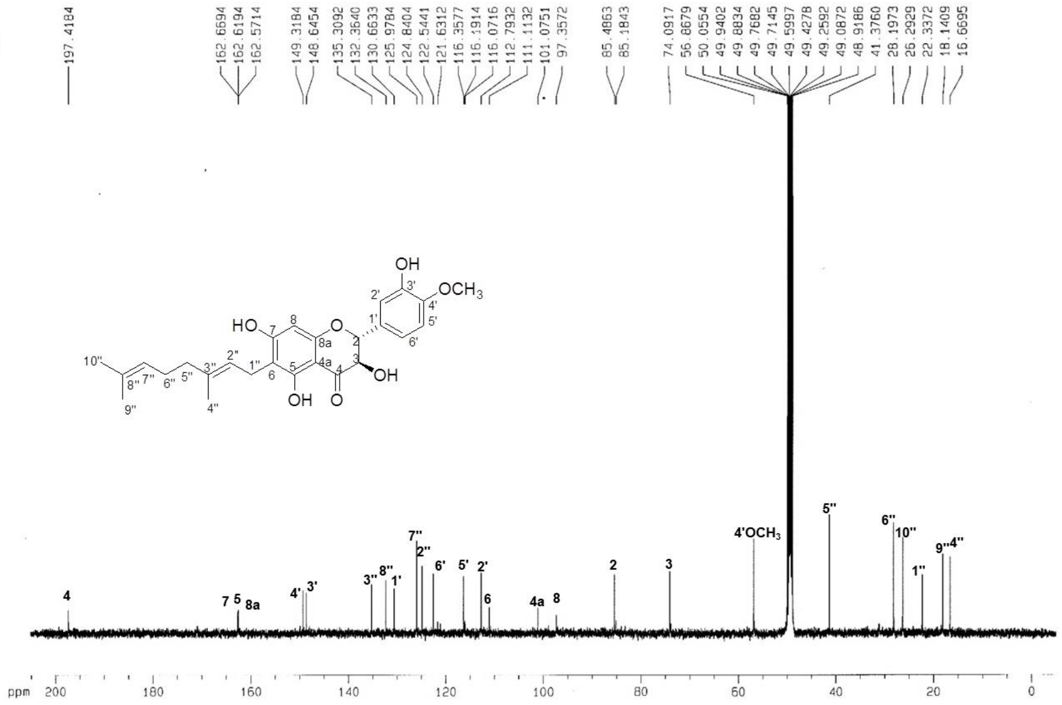

Figure 34. ^{13}C-NMR spectrum of compound **7** (125MHz, CD_3OD).

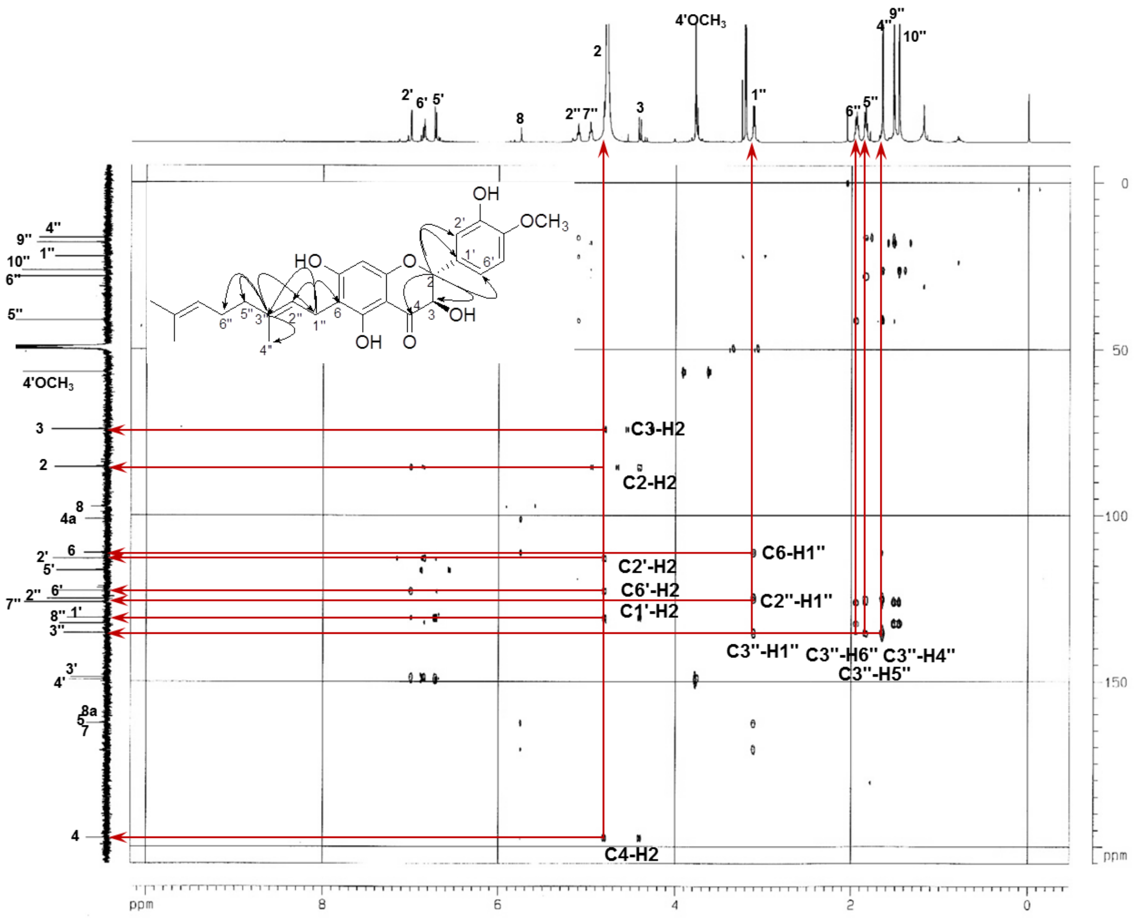

Figure 35. HMBC spectrum of compound **7**.

```
[ Elemental Composition ]
Data   :
Sample: -
Note  : -
Inlet  : Direct                              Ion Mode  : EI+
RT  : 1.24 min                               Scan#: 38
Elements  : C 100/1, H 100/1, O 20/1
Mass Tolerance       : 1000ppm, 1mmu if m/z < 1, 10mmu if m/z > 10
Unsaturation (U.S.)  : 5.0 - 30.0

Observed m/z  Int%    Err[ppm / mmu]     U.S.   Composition
  454.1993    100.0    +13.3 /   +6.0    21.0   C 33 H 26 O 2
                        +0.3 /   +0.2    12.0   C 26 H 30 O 7
```

Figure 36. HREIMS data of compound **7**.

Compound **1** (*Mimulone*); colorless powder; mp > 116℃; EIMS, *m/z* 408 [M]$^+$; HREIMS, *m/z* 408.1938 (calcd for $C_{25}H_{28}O_5$ 408.1937); ^{1}H-NMR (500 MHz, $CDCl_3$) δ 1.52 (3H, s, H-10), 1.60 (3H, s, H-9″), 1.73 (3H, s, H-4″), 1.99 (2H, m, H-5″), 2.02 (2H, m, H-6″), 2.70 (1H, dd, *J* = 2.6, 17.1 Hz, H-3b), 3.00 (1H, dd, *J* = 13.0, 17.1 Hz, H-3a), 3.26 (2H, d, *J* = 7.0 Hz, H-1″), 5.18 (1H, t, H-2″), 5.24 (1H, dd, *J* = 2.5, 13.0 Hz, H-2), 5.92 (1H, s, H-8), 6.79 (1H, s, H-5′), 6.80 (1H, s, H-3′), 7.22 (1H, s, H-6′), 7.24 (1H, s, H-2′). ^{13}C-NMR (125 MHz, $CDCl_3$) δ 16.6 (C-4″), 18.1 (C-10″), 21.5 (C-1″), 26.1 (C-9″), 26.8 (C-6″), 40.1 (C-5″), 43.6 (C-3), 79.2 (C-2), 96.1 (C-8), 103.3 (C-4a), 107.4 (C-6), 116.1 (C-3′), 116.1 (C-5′), 121.7 (C-2′), 124.1 (C-7′), 128.3 (C-2′), 128.3 (C-6′), 131.0 (C-1′), 132.5 (C-8″), 139.7 (C-3″), 156.6 (C-4′), 161.5 (C-8a), 161.7 (C-5), 164.5 (C-7), 196.7 (C=O, C-4).

Compound **2** (*3′-O-methyldiplacone*); colorless powder; mp > 103℃; EIMS, *m/z* 438 [M]$^+$; HREIMS, *m/z* 438.2035 (calcd for $C_{26}H_{30}O_6$ 438.2042); ^{1}H-NMR (500 MHz, CD_3OD) δ 1.47 (3H, s, H-10″), 1.53 (3H, s, H-9″), 1.65 (3H, s, H-4″), 1.84 (2H, m, H-5″), 1.95 (2H, m, H-6″), 2.45 (1H, dd, *J* = 3.1, 17.0 Hz, H-3b), 2.85 (1H, dd, *J* = 12.5, 17.0 Hz, H-3a), 3.08 (2H, d, *J* = 6.9 Hz, H-1″), 3.77 (3H, s, H-3′OCH_3), 4.99 (1H, t, H-7″), 5.08 (1H, dd, *J* = 3.1, 12.5 Hz, H-2), 5.14 (1H, t, H-2″), 5.62 (1H, s, H-8), 6.69 (1H, d, *J* = 8.0 Hz, H-5′), 6.78 (1H, dd, *J* = 1.8, 8.0 Hz, H-6′), 6.94 (1H, d, *J* = 1.8 Hz, H-2′). ^{13}C-NMR (125 MHz, CD_3OD) δ 16.7 (C-4″), 18.1 (C-10″), 22.6 (C-1″), 26.3 (C-9″), 28.3 (C-6″), 41.5 (C-5″), 44.5 (C-3), 56.8 (*OCH_3*-3′), 80.4 (C-2), 100.2 (C-8), 100.7 (C-4a), 111.5 (C-2′), 112.2 (C-6), 116.7 (C-5′), 120.7 (C-6′), 126.2 (C-2″), 126.2 (C-7″), 132.2 (C-8″), 132.8 (C-1′), 134.2 (C-3″), 149.7 (C-3′), 161.3 (C-4′), 162.9 (C-8a), 163.1 (C-5), 179.8 (C-7), 194.0 (C=O, C-4).

Compound **3** (*4′-O-methyldiplacone*); pale yellow powder; mp > 102℃; EIMS, *m*/*z* 438 $[M]^+$; HREIMS, *m*/*z* 438.2040 (calcd for $C_{26}H_{30}O_6$ 438.2042); ^{1}H-NMR (500 MHz, acetone–*d*$_6$); δ 1.58 (3H, s, H-10″), 1.64 (3H, s, H-9″), 1.79 (3H, s, H-4″), 1.98 (2H, m, H-5″), 2.07 (2H, m, H-6″), 2.74 (1H, dd, *J* = 3.0, 17.1 Hz, H-3b), 3.18 (1H, dd, *J* = 13.0, 17.1 Hz, H-3a), 3.30 (2H, d, *J* = 7.2 Hz, H-1″), 3.90 (3H, s, H-4′OCH_3), 5.10 (1H, t, H-7″), 5.29 (1H, t, H-2″), 5.41 (1H, dd, *J* = 2.8, 13.0 MHz, H-2), 6.07 (1H, s, H-8), 6.89 (1H, d, *J* = 8.1 Hz, H-5′), 7.00 (1H, dd, *J* = 1.9, 8.1 Hz, H-6′), 7.18 (1H, d, *J* = 1.9 Hz, H-2′). ^{13}C-NMR (125 MHz, acetone-*d*$_6$) δ 16.7 (C-4″), 18.2 (C-10″), 22.0 (C-1″), 26.3 (C-9″), 27.9 (C-6″), 40.9 (C-5″), 44.3 (C-3), 56.8 (OCH_3-4′), 80.6 (C-2), 95.8 (C-8), 103.6 (C-4a), 109.5 (C-6), 111.6 (C-2′), 116.1 (C-5′), 120.9 (C-6′), 123.9 (C-2″), 125.6 (C-7″), 131.9 (C-8″), 132.0 (C-1′), 135.5 (C-3″), 148.3 (C-3′), 148.8 (C-4′), 162.4 (C-8a), 162.8 (C-5), 165.2 (C-7), 197.7 (C=O, C-4).

Compound **4** (*6-Geranyl-3′,5,5′,7-tetrahydroxy-4′-methoxyflavanone*); colorless powder; mp > 108℃; EIMS, *m*/*z* 454 $[M]^+$; HREIMS, *m*/*z* 454.1992 (calcd for $C_{26}H_{30}O_7$ 454.1992); ^{1}H-NMR (500 MHz, acetone–*d*$_6$) δ 1.58 (3H, s, H-10″), 1.64 (3H, s, H-9″), 1.78 (3H, s, H-4″), 1.97 (2H, t, *J* = 7.0, 8.1 Hz, H-5″), 2.06 (2H, t, *J* = 4.4, 12.0 Hz, H-6″), 2.73 (1H, dd, *J* = 2.8, 17.1 Hz, H-3b), 3.16 (1H, dd, *J* = 13.0, 10.9 Hz, H-3a), 3.28 (2H, d, *J* = 7.1 Hz, H-1″), 3.86 (3H, s, H-4′OCH_3), 5.09 (1H, t, *J* = 6.2, 6.8 Hz, H-7″), 5.26 (1H, d, *J* = 10.9 Hz, H-2), 5.27 (1H, t, *J* = 7.0, 14.1 Hz, H-2″), 6.07 (1H, s, H-8), 6.72 (1H, s, H-6′), 6.73 (1H, s, H-2′); ^{13}C-NMR (125 MHz, acetone–*d*$_6$) δ 16.6 (C-4″), 18.1 (C-10″), 22.0 (C-1″), 26.2 (C-9″), 27.9 (C-5″), 40.9 (C-6″), 44.3 (C-3), 57.0 (C-4′OCH_3), 80.6 (C-2), 95.8 (C-8), 103.3 (C-2′), 103.5 (C-4a), 108.8 (C-6′), 109.5 (C-6), 123.9 (C-2″), 125.6 (C-7″), 131.3 (C-1′), 132.0 (C-8″), 135.4 (C-3″), 146.7 (C-3′, 5′), 149.4 (C-4′), 162.4 (C-8a), 162.7 (C-5), 165.3 (C-7), 197.7 (C-4).

Compound **5** (*3′-O-Methyl-5′-O-methyldiplacone*); colorless powder; mp > 78℃; EIMS, *m/z* 468 $[M]^+$; HREIMS, *m/z* 468.2151 (calcd for $C_{27}H_{32}O_7$ 468.2148). ^{1}H-NMR (500 MHz, acetone–d_6) δ 1.43 (3H, s, H-10″), 1.49 (3H, s, H-9″), 1.64 (3H, s, H-4″), 1.83 (2H, m, H-5″), 1.93 (2H, m, H-6″), 2.59 (1H, dd, J = 2.8, 17.1 Hz, H-3b), 3.06 (1H, dd, J = 13.0, 17.1 Hz, H-3a), 3.14 (2H, d, J = 7.1 Hz, H-1″), 3.72 (3H, s, H-3′OCH_3), 3.72 (3H, s, H-5′OCH_3), 4.95 (1H, t, H-7″), 5.13 (1H, t, H-2″), 5.26 (1H, dd, J = 2.8, 13.0, H-2), 5.93 (1H, s, H-8), 6.73 (1H, s, H-2′), 6.73 (1H, s, H-6′). ^{13}C-NMR (125 MHz, acetone–d_6) δ 16.6 (C-4″), 18.1 (C-10″), 22.0 (C-1″), 26.2 (C-9″), 27.9 (C-6″), 40.9 (C-5″), 44.4 (C-3), 57.2 (OCH_3-3′), 57.2 (OCH_3-5′), 80.9 (C-2), 95.8 (C-8), 103.5 (C-4a), 105.8 (C-2′), 105.8 (C-6′), 109.5 (C-6), 123.8 (C-2″), 125.6 (C-7″), 130.2 (C-1′), 132.0 (C-8″), 135.5 (C-3″), 137.7 (C-4′), 149.2 (C-3′), 149.2 (C-5′), 162.4 (C-8a), 162.8 (C-5), 165.1 (C-7), 197.7 (C=O, C-4).

Compound **6** (*3′-O-methyldiplacol*); pale yellow powder; mp > 140℃; EIMS, *m/z* 454 $[M]^+$; HREIMS, *m/z* 454.1990 (calcd for $C_{26}H_{30}O_7$ 454.1992); ^{1}H-NMR (500 MHz, acetone-d_6) δ 1.43 (3H, s, H-10″), 1.49 (3H, s, H-9″), 1.64 (3H, s, H-4″), 1.82 (2H, m, H-5″), 1.94 (2H, m, H-6″), 3.15 (2H, d, J = 7.1 Hz, H-1″), 3.74 (3H, s, H-3′OCH_3), 4.53 (1H, d, J = 11.6 Hz, H-3), 4.91 (1H, d, J = 11.6 Hz, H-2), 4.95 (1H, t, H-7″), 5.12 (1H, t, H-2″), 5.91 (1H, s, H-8), 6.74 (1H, d, J = 8.0 Hz, H-2′), 6.89 (1H, dd, J = 1.7, 8.0 Hz, H-6′), 7.07 (1H, d, J = 1.7 Hz, H-5′). ^{13}C-NMR (125 MHz, acetone-d_6) δ 16.6 (C-4″), 18.1 (C-10″), 22.0 (C-1″), 26.2 (C-9″), 27.8 (C-6″), 40.9 (C-5″), 56.8 (OCH_3-3′), 80.8 (C-3), 85.1 (C-2), 96.0 (C-8), 105.8 (C-4a), 109.8 (C-6), 112.8 (C-5′), 115.8 (C-2′), 122.5 (C-6′), 123.8 (C-2″), 125.6 (C-7″), 130.2 (C-1′), 132.0 (C-8″), 135.6 (C-3″), 148.4 (C-4′), 148.6 (C-3′), 149.8 (C-8a), 162.2 (C-5), 165.9 (C-7), 198.6 (C=O, C-4).

Compound **7** (*4′-O-methyldiplacol*); pale yellow powder; mp > 140°C; EIMS, *m/z* 454 $[M]^+$; HREIMS, *m/z* 454.1993 (calcd for $C_{26}H_{30}O_7$ 454.1992); ^{1}H-NMR (500 MHz, CD_3OD) *δ* 1.46 (3H, s, H-10″), 1.52 (3H, s, H-9″), 1.67 (3H, s, H-4″), 1.84 (2H, m, H-5″), 1.94 (2H, m, H-6″), 3.11 (2H, d, *J* = 7.1 Hz, H-1″) , 3.78 (3H, s, H-4′OCH_3), 4.41 (1H, d, *J* = 10.8 Hz, H-3), 4.82 (1H, d, *J* = 10.8 Hz, H-2), 5.10 (1H, t, H-7″), 5.12 (1H, t, H-2″), 5.75 (1H, s, H-8), 6.73 (1H, d, *J* = 8.1 Hz, H-5′), 6.85 (1H, dd, *J* = 1.8, 8.1 Hz, H-6′), 7.00 (1H, d, *J* = 1.8 Hz, H-2′). ^{1} ^{3}C-NMR (125 MHz, CD_3OD) *δ* 16.7 (C-4″), 18.1 (C-9″), 22.3 (C-1″), 26.3 (C-10″), 28.2 (C-6″), 41.4 (C-5″), 56.9 (*OCH_3*-4′), 74.1 (C-3), 85.5 (C-2), 97.4 (C-8), 101.1 (C-4a), 111.1 (C-6), 112.8 (C-2′), 116.4 (C-5′), 122.5 (C-6′), 124.8 (C-2″), 126.0 (C-7″), 130.7 (C-1′), 132.4 (C-8″), 135.3 (C-3″), 148.6 (C-3′), 149.3 (C-4′), 162.5 (C-8a), 162.6 (C-5), 162.7 (C-7), 197.4 (C=O, C-4).

Compound **8** (*6-Geranyl-3,3′,5,5′,7-pentahydroxy-4′-methoxyflavane*); white needles; EIMS, *m/z* 470 [M]+; HREIMS, *m/z* 470.1943 (calcd for $C_{26}H_{30}O_8$ 470.1941); ^{1}H-MR (500 MHz, acetone–d_6) *δ* 1.44 (3H, s, H-10″), 1.49 (3H, s, H-9″), 1.64 (3H, s, H-4″), 1.83 (2H, t, *J* = 7.0, 8.2 Hz, H-6″), 1.92 (2H, d, *J* = 2.2 Hz, H-5″), 3.15 (2H, d, *J* = 7.1 Hz, H-1″), 3.71 (2H, s, H-4′OCH_3), 4.49 (1H, d, *J* = 11.4 Hz, H-3), 4.84 (1H, d, *J* = 11.4 Hz, H-2), 4.96 (1H, dd, *J* = 5.6, 7.0 Hz, H-7″), 5.13 (1H, t, *J* = 1.1, 6.1 Hz, H-2″), 5.91 (2H, s, H-8), 6.62 (2H, d, *J* = 4.2 Hz, H-2′, 6′); ^{13}C-NMR (125 MHz, acetone–d_6) *δ* 16.6 (C-4″), 18.1 (C-10″), 22.0 (C-1″), 26.2 (C-9″), 27.8 (C-6″), 40.9 (C-5″), 57.0 (C-4′OCH_3), 73.7 (C-3), 85.3 (C-2), 96.0 (C-8), 101.8 (C-4a), 104.9 (C-6′), 109.8 (C-6), 110.2 (C-2′), 123.8 (C-2″), 125.6 (C-7″), 129.5 (C-1′), 132.0 (C-8″), 135.5 (C-3′), 135.7 (C-3″), 146.5 (C-5′), 149.2 (C-4′), 162.2 (C-8a), 162.4 (C-5), 165.9 (C-7), 198.64 (C-4).

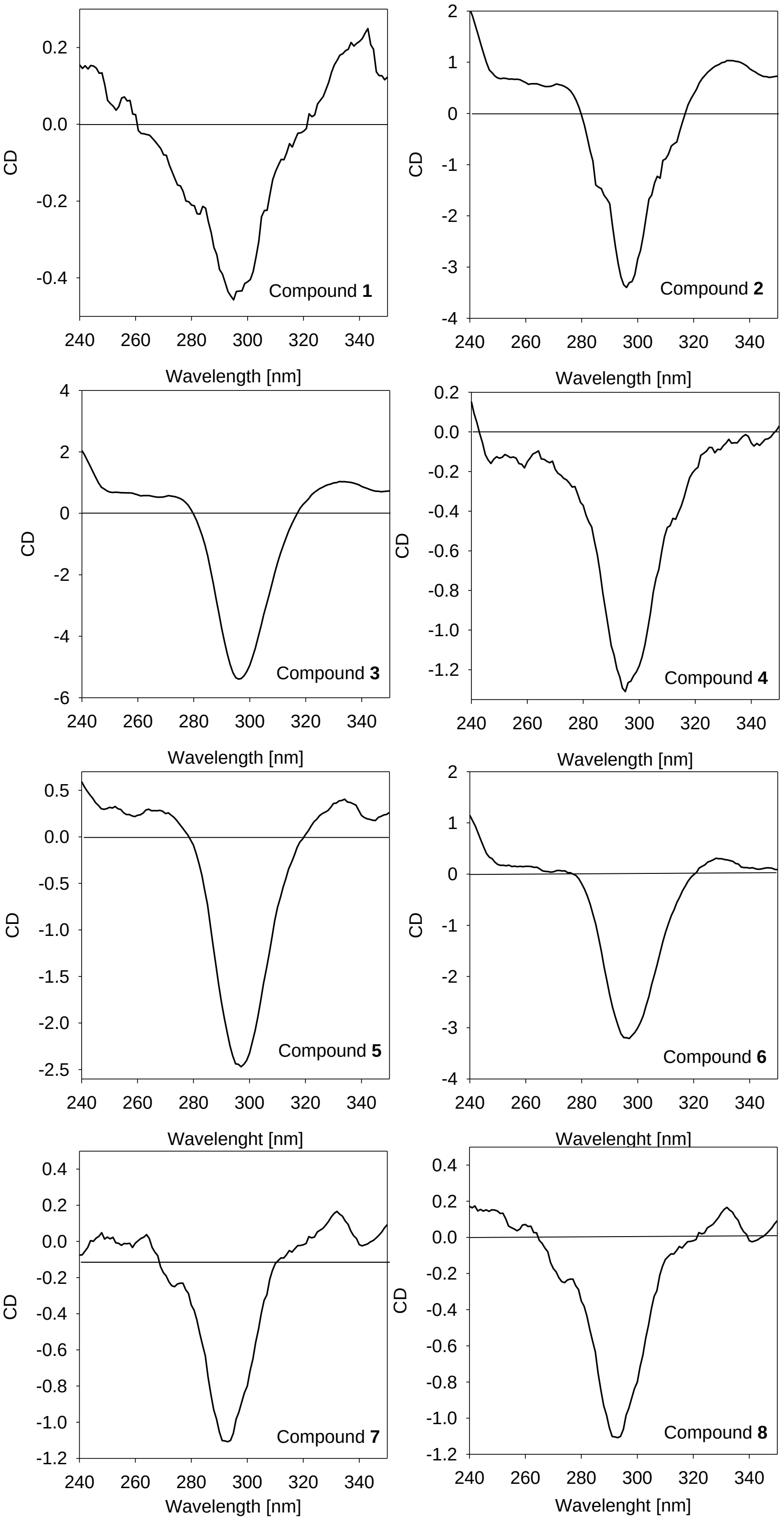

Figure 37. CD spectrum of compounds **1-8**.

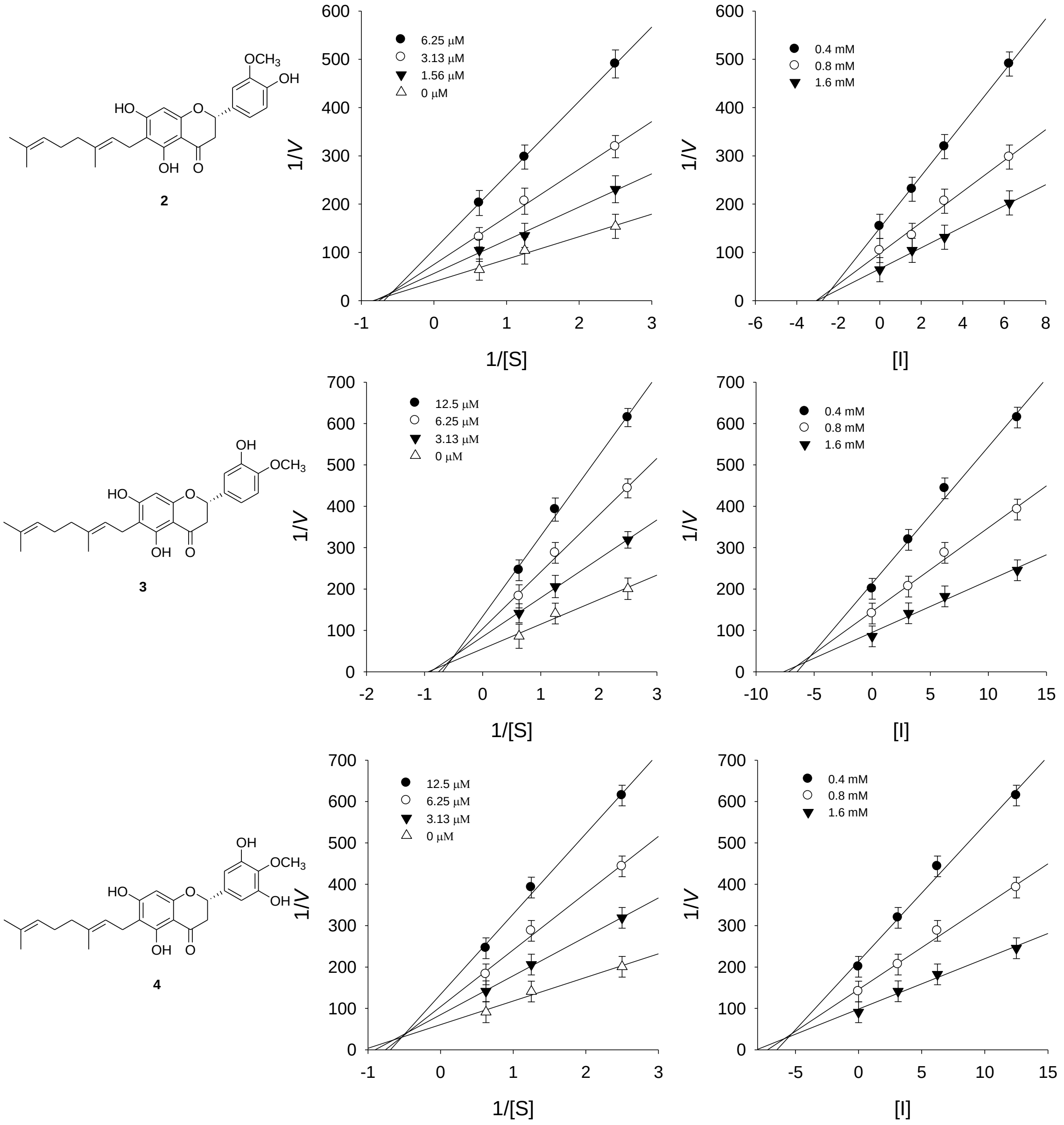

Figure 38. Lineweaver-Burk plots for the inhibition of PTP1B activities by compound **2-4** (left). Dixon plots for the inhibition of compounds **2-4** (right).

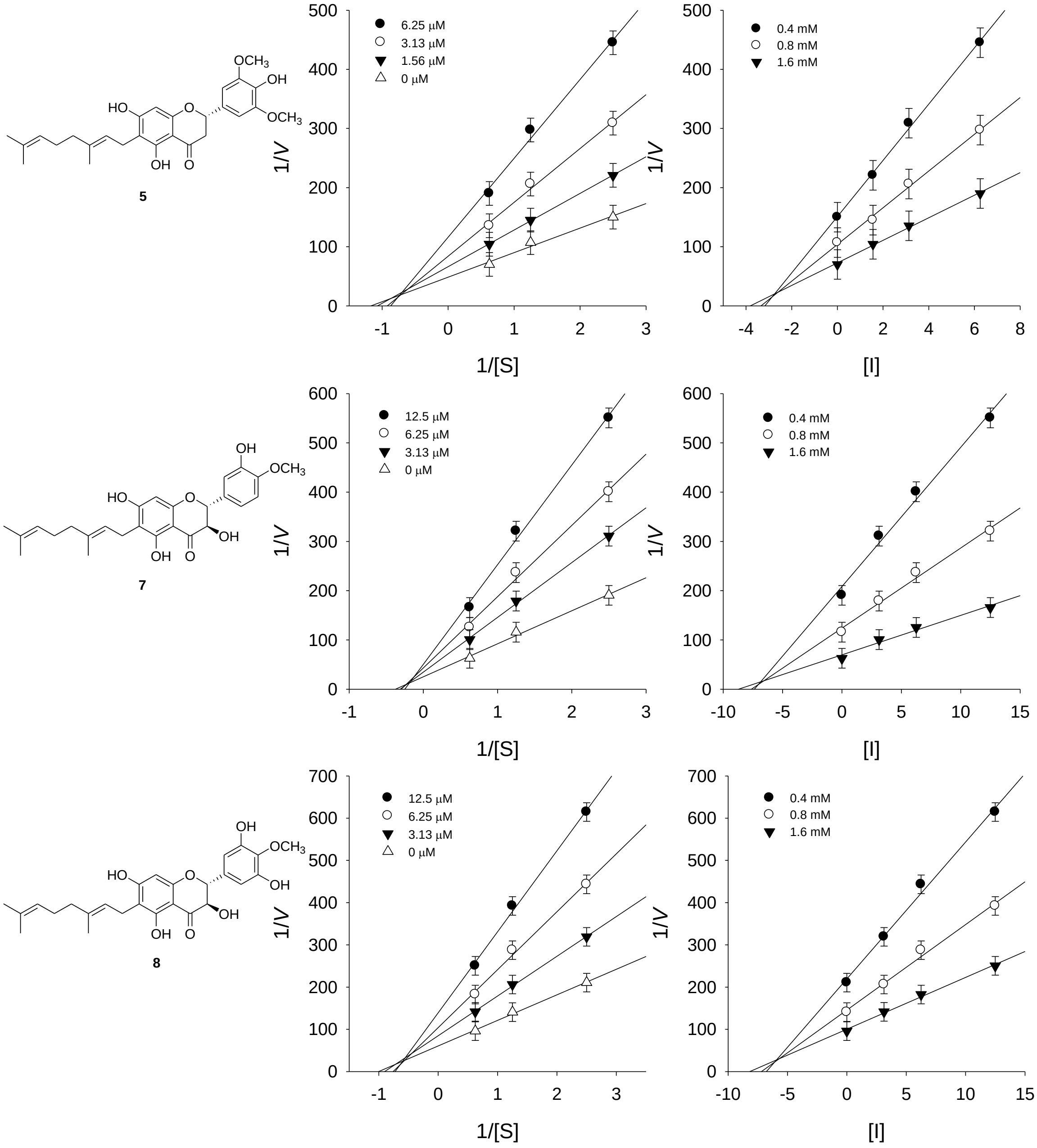

Figure 39. Lineweaver-Burk plots for the inhibition of PTP1B activities by compound **5, 7, 8** (left). Dixon plots for the inhibition of compounds **5**, **7**, **8** (right).

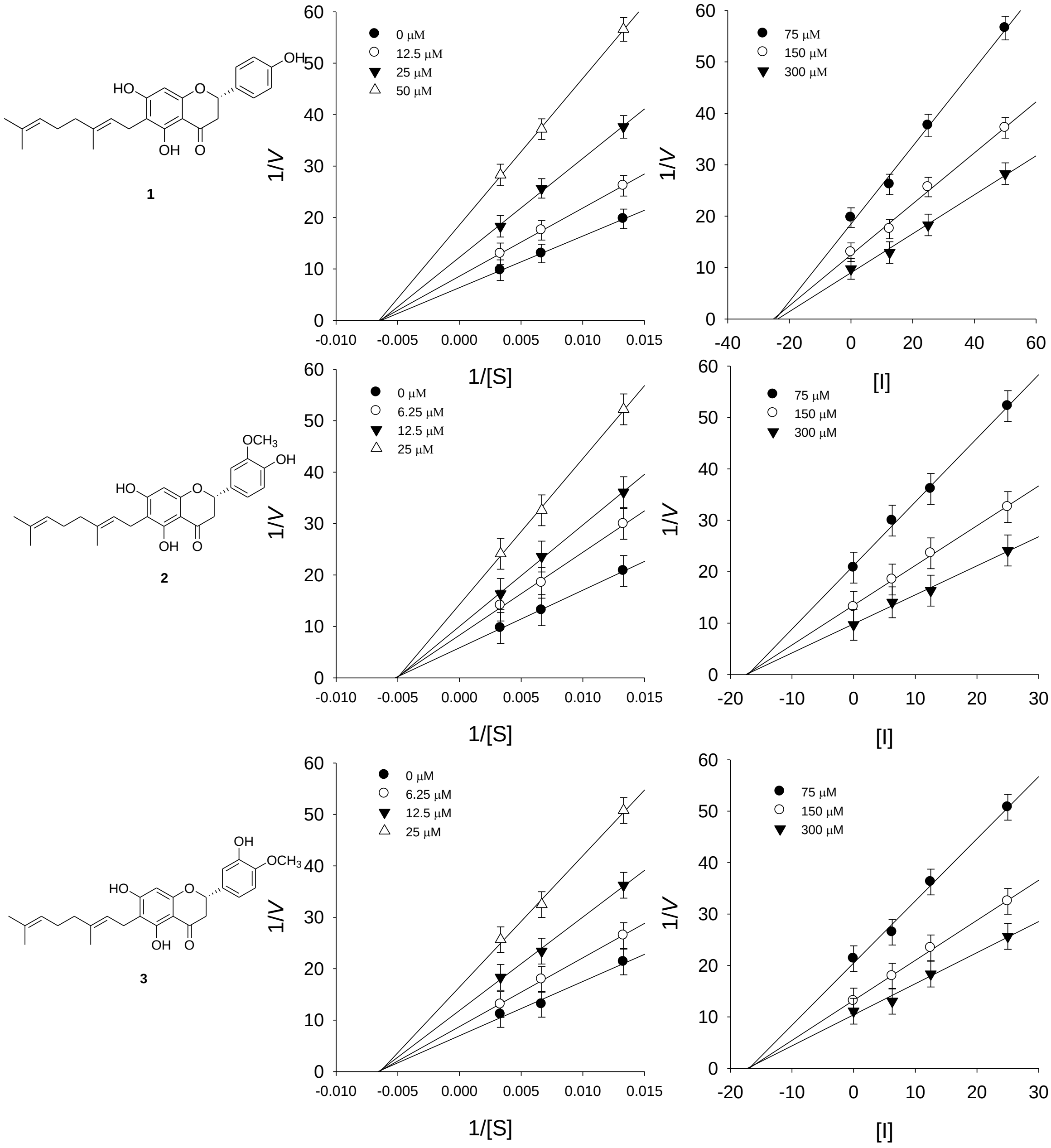

Figure 40. Lineweaver-Burk plots for the inhibition of α-glucosidase activities by compound **1**-**3** (left). Dixon plots for the inhibition of compounds **1**-**3** (right).

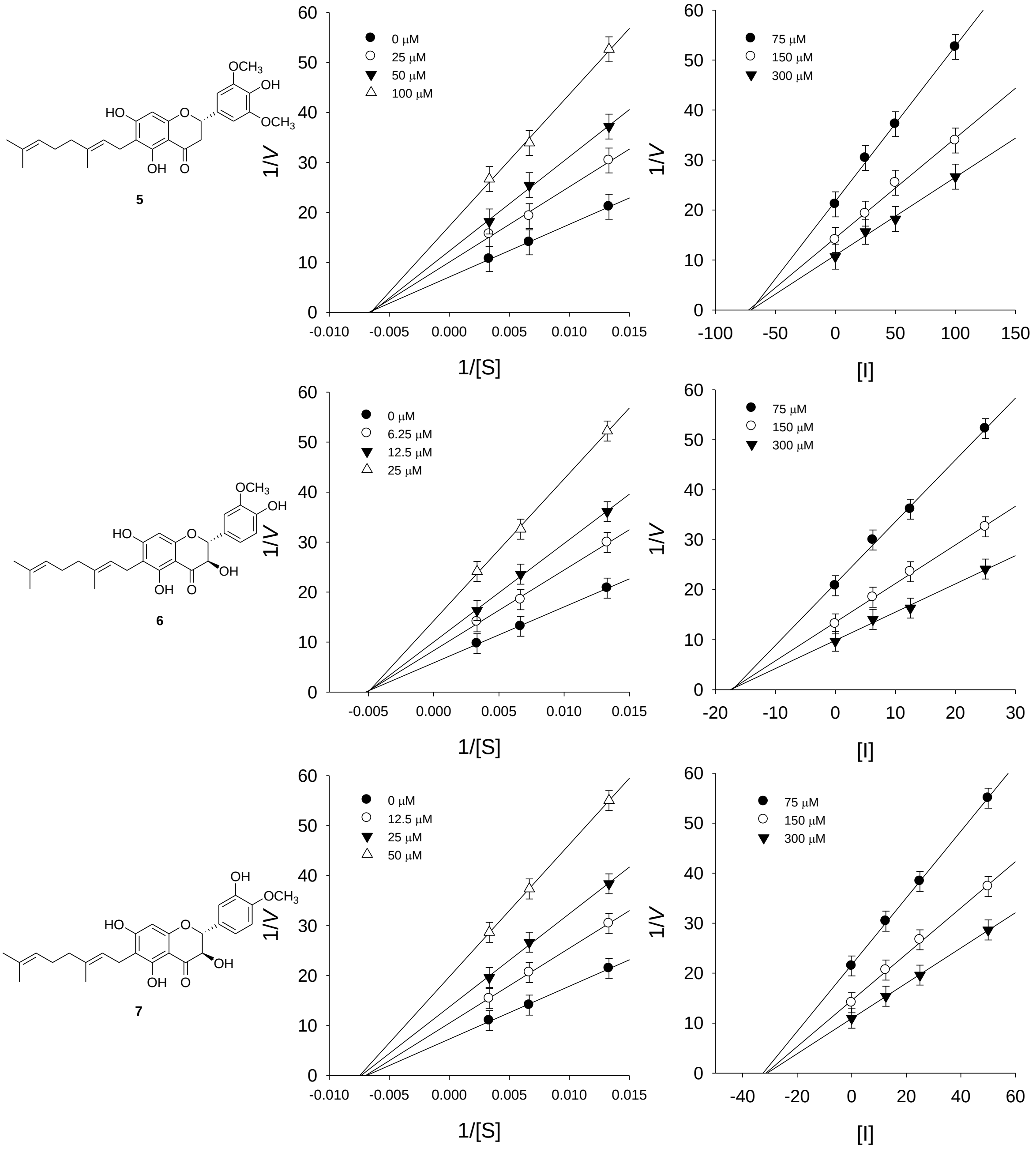

Figure 41. Lineweaver-Burk plots for the inhibition of α-glucosidase activities by compound **5-7** (left). Dixon plots for the inhibition of compounds **5-7** (right).

Table 1. Inhibitory effect of isolated compounds **1**−**8** on PTP1B.

Compounds	IC_{50} value (μM)	Inhibition mode	K_I (μM)	K_{IS} (μM)
**1**	1.9 ± 0.1	Mixed Type Ⅰ	0.4 ± 0.06	2.2 ± 0.1
**2**	3.9 ± 0.3	Mixed Type Ⅰ	2.6 ± 0.1	3.7 ± 0.2
**3**	7.8 ± 0.6	Mixed Type Ⅰ	5.7 ± 0.3	10.1 ± 0.4
**4**	5.9 ± 0.4	Mixed Type Ⅰ	5.5 ± 0.3	11.2 ± 0.4
**5**	3.8 ± 0.3	Mixed Type Ⅰ	2.8 ± 0.2	4.6 ± 0.2
**6**	4.9 ± 0.5	Mixed Type Ⅰ	3.5 ± 0.2	5.2 ± 0.2
**7**	8.2 ± 0.6	Mixed Type Ⅰ	6.9 ± 0.4	13.7 ± 0.6
**8**	6.6 ± 0.5	Mixed Type Ⅰ	5.9 ± 0.3	10.3 ± 0.5
